# Perfusion imaging findings, and outcomes between computed tomography perfusion selected basilar artery occlusion and anterior circulation stroke patients undergoing endovascular treatment

**DOI:** 10.1371/journal.pone.0353204

**Published:** 2026-07-14

**Authors:** Xiaofeng Cai

**Affiliations:** Department of Neurology, Centre for Rehabilitation Medicine, Zhejiang Provincial People’s Hospital, Affiliated People’s Hospital, Hangzhou Medical College , Hangzhou, Zhejiang, China; Fondazione Policlinico Universitario Agostino Gemelli IRCCS, ITALY

## Abstract

**Background:**

Basilar artery occlusion (BAO) is a severe form of ischemic stroke associated with high morbidity and mortality. Whether BAO patients derive similar benefits from CT perfusion (CTP)-guided endovascular treatment (EVT) as those with anterior circulation stroke (ACS) remains unclear.

**Aim:**

This study aimed to compare the efficacy and safety of EVT based on computed tomography (CT) perfusion between patients with BAO and those with ACS.

**Methods:**

This analysis was conducted using data from a prospective multicenter Registry on International Stroke Perfusion Imaging. A total of 256 patients with 36 BAO and 220 ACS who received CTP-guided EVT were included. Propensity score matching (PSM) in a 1:2 ratio was performed to adjust for baseline imbalances. Multivariate logistic regression was used to identify independent predictors of mRS 3–6 at 90 days as poor functional outcome and mortality.

**Results:**

Prior to matching, BAO patients had higher baseline NIHSS scores (24.0 ± 9.8 vs. 15.2 ± 6.4, p < 0.001), smaller ischemic core volumes (8.7 ± 11.4 mL vs. 33.2 ± 38.9 mL, p < 0.001), and higher mismatch ratios (0.9 ± 0.1 vs. 0.7 ± 0.2, p < 0.001) than ACS patients. After matching, BAO patients had significantly shorter door-to-reperfusion time (3.2 ± 1.1 vs. 4.3 ± 2.9 hours, p = 0.007) and lower rates of hemorrhagic transformation (19.4% vs. 48.6%, p = 0.006). Multivariate analysis identified baseline NIHSS score (OR = 1.052, 95% CI: 1.013–1.092, p = 0.009) and ASPECT score (OR = 0.813, 95% CI: 0.682–0.969, p = 0.021) as independent predictors of poor outcome, while age (OR = 1.037, 95%CI: 1.006–1.069, p = 0.020) and baseline NIHSS score (OR = 1.070, 95% CI: 1.024–1.119, p = 0.002) were independent predictors of mortality. No significant differences were observed in 90-day functional independence (mRS 0–2) or mortality between BAO group and ACS group.

**Conclusions:**

Despite presenting with more severe neurological deficits, BAO patients treated with CTP-guided EVT achieved comparable functional outcomes and mortality to ACS patients, with shorter reperfusion times and fewer hemorrhagic complications. CTP-based selection may facilitate effective EVT in BAO.

## Introduction

Endovascular treatment (EVT) for recanalizing large vessel occlusions (LVO) has become a critical component of standard care for managing patients with acute ischemic stroke (AIS). Several randomized clinical trials have demonstrated the safety and efficacy of EVT in select patients with proximal occlusions in anterior circulation stroke (ACS) [1–5]. However, about 5% of all patients who undergo thrombolysis for stroke have basilar artery occlusion (BAO), which is among the most severe medical conditions, with very high fatality rates even after reperfusion achieved by EVT [6,7].

Two multicenter RCTs failed to demonstrate a significant benefit of EVT over best medical management (BMM), though methodological limitations were later identified [8,9]. Emerging evidence from the Endovascular thrombectomy for acute basilar artery occlusion (ATTENTION) and Endovascular thrombectomy for acute basilar artery occlusion (BAOCHE) trials has established the superiority of EVT in BAO patients. Crucially, subgroup analyses indicated that the therapeutic benefit is predominantly driven by patients with moderate-to-severe baseline stroke severity (NIHSS ≥10), while patients with mild symptoms (NIHSS <10) did not demonstrate significant benefit [10–12].

This study aimed to investigate the efficacy and safety of EVT based on computed tomography (CT) perfusion in patients with BAO compared to those with ACS. Furthermore, we aimed to analyze the prognostic factors associated with poor outcomes and mortality, with the goal of providing valuable insights into the optimal management of these patients.

## Methods

### Study population

Data for this study were obtained from our site within the International Stroke Perfusion Imaging Registry, with central ethics approval from the Hunter New England Health District Ethics Committee (11/08/17/4.01). We retrospectively reviewed consecutive cases of acute large artery occlusion (LAO) within 24 hours of symptom onset in patients who underwent endovascular treatment (EVT) with computed tomography perfusion (CTP) scans. The dates were accessed for research purposes in Jan. 2026. Patients were included if they met the following criteria: (1) internal carotid artery (ICA), middle cerebral artery (MCA), anterior cerebral artery (ACA), or basilar artery (BA) occlusion confirmed on pretreatment four-dimensional CT angiography (4D-CTA) reconstructed from CTP; and (2) pre-stroke modified Rankin Scale (mRS) score ≤ 2. Patients were excluded if they had poor image quality owing to motion artifacts or were unable to undergo follow-up CT before discharge.

Patients were divided into two groups based on the occlusion location: the basilar artery occlusion (BAO) group and the anterior circulation stroke (ACS) group, which included patients with ICA, MCA, or ACA occlusions. Clinical and neuroimaging characteristics were evaluated, including patient age, sex, medical history (hypertension, diabetes mellitus, atrial fibrillation [AF], tobacco use, and hypercholesterolemia), baseline National Institutes of Health Stroke Scale (NIHSS) score, pre-stroke mRS score, Alberta Stroke Program Early CT Score (ASPECT), stroke etiology (cardioembolic vs. others), and treatment details (intravenous recombinant tissue plasminogen activator [rt-PA] use, device type, and procedural time intervals).

The protocols of the study had been approved by the local ethics committee of Zhejiang Provincial People’s Hospital. All clinical investigation has been conducted according to the principles expressed in the Declaration of Helsinki.

### Imaging protocol

**Fig 1:**
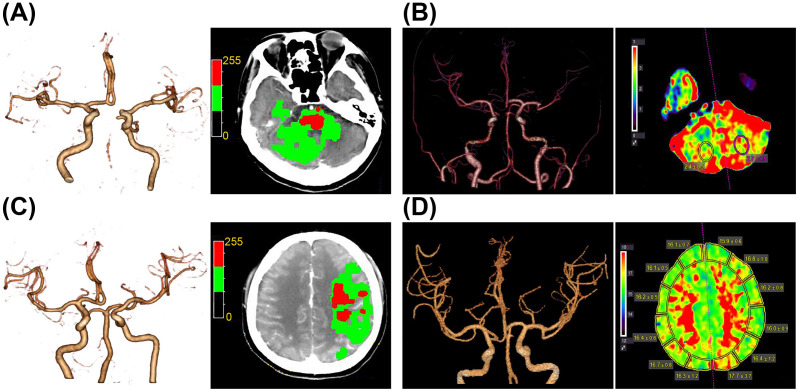
Imaging manifestations of BAO and ACS patient before and after CTP guided EVT treatment. (A) Preoperative CTA showed occlusion of the basilar artery and CTP showed low perfusion in the basal artery supply area, with the red area indicating the core infarct zone. (B) Postoperative CTA indicated recanalization of the basilar artery and CTP indicated the recovery of blood flow in the basal artery supply area. (C) Preoperative CTA showed occlusion of the upper trunk of the M1 segment of the left middle cerebral artery and CTP showed hypoperfusion in the left middle cerebral artery supply area, with the red area indicating the core infarct zone. (D) Postoperative CTA showed recanalization of the left middle cerebral artery and CTP indicated the recovery of blood flow in the left cerebral blood supply area.

All patients underwent baseline non-contrast CT (NCCT) and CTP prior to reperfusion treatment, as well as follow-up NCCT before discharge. CT imaging was performed using a 320-detector row, 640-slice cone-beam multidetector CT scanner (Aquilion One, Toshiba Medical Systems). Whole-brain NCCT was acquired in a single rotation (detector width: 16 cm). CTP was performed following administration of 50 mL of contrast agent (Ultravist 370; Bayer HealthCare, Berlin, Germany) injected intravenously at a rate of 6 mL/s, followed by 50 mL of saline (acquisition parameters: 120 kV, 128 mA; scanning coverage = 240 mm; scanning width = 5 mm). A pulsed full-rotation scan protocol with 18 time points acquired over 60 seconds and a total pulse image acquisition time of 9.5 seconds was employed, commencing 7 seconds after contrast injection. The follow-up NCCT scanning protocol was identical to that used for baseline NCCT. The imaging manifestations of BAO and ACS patient before and after CTP guided EVT treatment were shown in Fig 1.

Assessment of Hypoperfusion and Ischemic Core Volume CTP data were postprocessed using MIStar software (Apollo Medical Imaging Technology, Melbourne, Australia). The hypoperfusion volume was defined using a delay time (DT) threshold of >3 seconds. The ischemic core volume was calculated using a relative cerebral blood flow (rCBF) threshold of <30% [13]. The penumbra volume was defined as the hypoperfusion volume minus the ischemic core volume. The mismatch ratio was calculated as the penumbra volume divided by the total hypoperfusion volume, representing the proportion of potentially salvageable tissue within the total hypoperfused region. **The present study applies established perfusion parameters but does not aim to validate or optimize CTP thresholds for posterior circulation stroke.**

### Outcome assessment

The primary outcomes were poor functional outcome (mRS 3–6) at 90 days and mortality. The NIHSS score was evaluated at three time points: baseline, 24 hours after admission, and 14 days. The Modified Rankin Scale (mRS) score was used to assess clinical outcomes at 90 days after onset. An mRS score of 0–2 was designated as a good outcome, and an mRS score of 3–6 was designated as a poor outcome. In-hospital mortality was defined as death occurring during hospitalization or within 24 hours after discharge. For 90-day outcome assessment, mortality was defined as death (mRS score of 6) occurring at any time within 90 days of symptom onset. Hemorrhagic transformation (HT) was classified as hemorrhagic infarction (HI) or parenchymal hemorrhage (PH) according to the European Cooperative Acute Stroke Study (ECASS) definition [14]. All outcomes, including HT, and functional outcomes, were adjudicated by a clinician with 10 years of experience in stroke management.

### Statistical analysis

Statistical analyses were performed using Python version 3.10.19. Demographic, baseline clinical, and imaging characteristics were summarized using descriptive statistics. Continuous variables were expressed as mean ± standard deviation (SD) and compared between groups using independent-samples t-tests for normally distributed data or the Mann–Whitney U test for non-normally distributed data. Categorical variables were expressed as frequencies and percentages and compared using the chi-square test or Fisher’s exact test, as appropriate. Comparisons were performed in both the primary (unmatched) cohort and the PSM cohort. Univariate logistic regression analysis was performed to identify potential predictors of poor outcome (mRS 3–6 at 90 days) and mortality among all patients with LAO who underwent EVT. Collinearity among predictor variables was assessed prior to multivariate modeling (Supplementary Fig. 2). To account for potential confounding factors between the BAO and ACS groups, propensity score matching (PSM) was performed. Propensity scores were estimated using a logistic regression model incorporating the following baseline covariates: age, sex, hypertension, diabetes mellitus, atrial fibrillation, tobacco use, baseline mRS score, ASPECT, stroke etiology, and hypercholesterolemia. Each BAO patient was matched to two ACS patients (1:2 ratio) using nearest-neighbor matching without replacement. The caliper width was set at 0.2 standard deviations of the logit of the propensity score. Covariate balance after matching was assessed by comparing baseline characteristics between the two groups. Variables with P < 0.1 in univariate analysis were entered into a multivariate logistic regression model. Results were reported as odds ratios (ORs) with 95% confidence intervals (CIs). Statistical significance was set at P < 0.05 for multivariate analyses.

The protocols of the study had been approved by the local ethics committee of Zhejiang Provincial People’s Hospital Results.

## Results

### Patient characteristics

**Fig 2:**
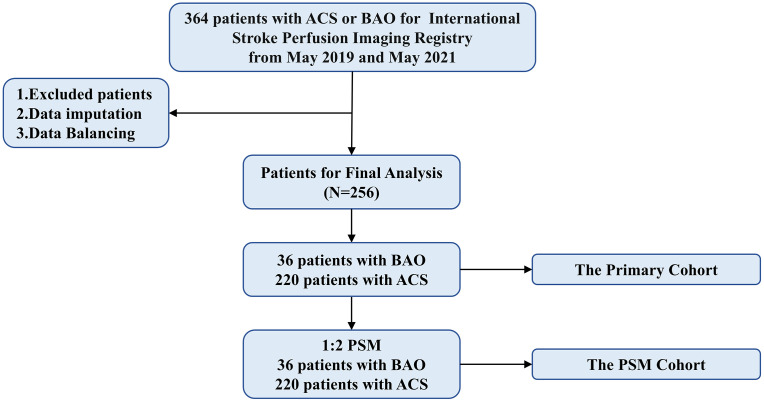
The flowchart of patients’ enrollment.

The study flowchart is shown in Fig 2. A total of 256 patients (mean age 67.8 ± 14.0 years, 50.4% male) met the inclusion criteria and were enrolled. Among them, 36 (14.1%) patients had BAO and 220 (85.9%) patients had ACS. Baseline clinical and neuroimaging characteristics of the primary cohort are presented in Table 1. There were 2 ACA, 77 ICA, and 141 MCA in ACS group. There was no no significant difference in their clinical characteristics (S1 Table). Therefore, classifying these patients into the ACS group and the analysis results of BAO are reliable.

**Table 1:** Baseline clinical characteristics of patients.

Variables	Primary Cohort			1:2 PSM Cohort		
	BAO (n = 36)	ACS (n = 220)	P	BAO (n = 36)	ACS (n = 72)	P
Age, Mean ± SD	64.9 ± 13.0	68.3 ± 14.1	0.107	64.9 ± 13.0	68.1 ± 15.5	0.213
Sex			1.000			1.000
Male	18	111		18	36	
Female	18	109		18	36	
Hypertension			0.953			0.942
Yes	24	142		24	50	
No	12	78		12	22	
Diabetes mellitus			0.097			0.691
Yes	10	33		10	16	
No	26	187		26	56	
AF history			0.198			1.000
Yes	9	83		9	19	
No	27	137		27	53	
Tobacco use			0.512			0.330
Yes	6	51		6	6	
No	30	169		30	66	
Hypercholesterolemia			**0.009**			0.574
Yes	5	6		5	6	
No	31	214		31	66	
Baseline NIHSS Score, Mean ± SD	24.0 ± 9.8	15.2 ± 6.4	**<0.001**	24.0 ± 9.8	19.7 ± 6.9	**<0.001**
Baseline mRS Score, Mean ± SD	0.2 ± 0.4	0.4 ± 1.1	0.716	0.2 ± 0.4	0.2 ± 0.7	0.335
APECT, Mean ± SD	7.6 ± 2.0	7.9 ± 1.6	0.445	7.6 ± 2.0	7.8 ± 1.8	0.660
Stroke etiology			1.000			0.924
Cardioembolic	30	182		30	62	
Others	6	38		6	10	
Ischemic Core Volume, Mean ± SD	8.7 ± 11.4	33.2 ± 38.9	**<0.001**	8.7 ± 11.4	39.1 ± 42.6	**<0.001**
Hypoperfusion Volume, Mean ± SD	100.9 ± 42.5	120.9 ± 71.6	0.176	100.9 ± 42.5	130.7 ± 68.2	0.051
Mismatch Volume, Mean ± SD	92.2 ± 40.5	87.8 ± 61.0	0.275	92.2 ± 40.5	91.6 ± 54.0	0.835
Mismatch ratio, Mean ± SD	0.9 ± 0.1	0.7 ± 0.2	**<0.001**	0.9 ± 0.1	0.7 ± 0.2	**<0.001**

In the primary cohort, patients with BAO tended to be younger than those with ACS, although the difference did not reach statistical significance (64.9 ± 13.0 vs. 68.3 ± 14.1 years, P = 0.107). There were no significant differences between the two groups in sex distribution, or in the prevalence of hypertension, atrial fibrillation, or tobacco use. Diabetes mellitus tended to be more common in the BAO group, though this difference was not statistically significant (27.8% vs. 15.0%, P = 0.097). Hypercholesterolemia was significantly more frequent in BAO patients compared with ACS patients (13.9% vs. 2.7%, P = 0.009). Cardioembolic etiology was similarly distributed between the two groups (83.3% vs. 82.7%, P = 1.000). The mean baseline NIHSS score was significantly higher in the BAO group compared with the ACS group (24.0 ± 9.8 vs. 15.2 ± 6.4, P < 0.001). There were no significant differences in pre-stroke mRS score or ASPECT between the two groups.

Regarding baseline neuroimaging, the mean ischemic core volume was significantly smaller in BAO patients compared with ACS patients (8.7 ± 11.4 mL vs. 33.2 ± 38.9 mL, P < 0.001). The hypoperfusion volume and mismatch volume did not differ significantly between the two groups. However, the mismatch ratio was significantly higher in BAO patients than in ACS patients (0.9 ± 0.1 vs. 0.7 ± 0.2, P < 0.001), indicating a greater proportion of salvageable tissue relative to the total hypoperfused region in the posterior circulation.

### Propensity score–matched cohort

After 1:2 PSM, 36 BAO patients were matched with 72 ACS patients, yielding a well-balanced cohort (Table 1). In the PSM cohort, baseline demographic characteristics—including age (64.9 ± 13.0 vs. 68.1 ± 15.5 years, P = 0.213), sex distribution, and comorbidities—were comparable between the two groups. The baseline NIHSS score remained significantly higher in the BAO group (24.0 ± 9.8 vs. 19.7 ± 6.9, P < 0.001). The ischemic core volume remained significantly smaller in BAO patients (8.7 ± 11.4 mL vs. 39.1 ± 42.6 mL, P < 0.001), while the hypoperfusion volume showed a trend toward being smaller in BAO patients (100.9 ± 42.5 mL vs. 130.7 ± 68.2 mL, P = 0.051). The mismatch ratio remained significantly higher in the BAO group (0.9 ± 0.1 vs. 0.7 ± 0.2, P < 0.001).

### Procedural characteristics

Treatment and procedural details are shown in Table 2. In the primary cohort, stent retriever thrombectomy was used as the sole device in the majority of patients in both groups (72.2% in BAO and 85.9% in ACS). Combined stent retriever and aspiration was more frequently used in BAO patients (16.7% vs. 5.9%), and the overall distribution of device use trended toward a difference between the two groups (P = 0.054). Rates of intravenous rt-PA administration were comparable between the BAO and ACS groups (16.7% vs. 20.9%, P = 0.717).

**Table 2:** Treatment and outcome characteristics of patients.

Variables	Primary Cohort			1:2 PSM Cohort		
	BAO (n = 36)	ACS (n = 220)	P	BAO (n = 36)	ACS (n = 72)	P
**Treatment Method, n(%)**						
Intravenous rt-PA	6(16.7%)	46(20.9%)	0.717	6(16.7%)	10(13.9%)	0.924
Device Use			0.054			0.086
Stent retriever	26(72.2%)	189(85.9%)		26(72.2%)	60(83.3%)	
Stent + Aspiration	6(16.7%)	13(5.9%)		6(16.7%)	3(4.2%)	
Aspiration only	4(11.1%)	18(8.2%)		4(11.1%)	9(12.5%)	
**Treatment Times, Mean ± SD**						
Onset-Door Time	5.1 ± 4.1	6.5 ± 5.6	0.231	5.1 ± 4.1	5.8 ± 4.9	0.632
Onset-Needle Time(rt-PA Only)	2.5 ± 1.6	3.5 ± 5.7	0.864	2.5 ± 1.6	2.2 ± 0.9	0.871
Onset-Puncture Time	7.2 ± 4.1	9.0 ± 6.1	0.169	7.2 ± 4.1	8.3 ± 5.3	0.492
Onset-Reperfusion Time	8.3 ± 4.3	10.5 ± 6.3	0.072	8.3 ± 4.3	10.1 ± 5.8	0.175
Door-Reperfusion Time	3.2 ± 1.1	4.0 ± 2.6	0.108	3.2 ± 1.1	4.3 ± 2.9	**0.007**
**Complications, n (%)**						
Hemorrhagic Transformation	7(19.4%)	96(43.6%)	**0.010**	7(19.4%)	35(48.6%)	**0.006**
Parenchymal Hemorrhage	4(11.1%)	46(20.9%)	0.255	4(11.1%)	19(26.4%)	0.083
**Clinical Outcomes**						
NIHSS at 24h, Mean±SD	19.4 ± 13.7	14.3 ± 11.1	**0.046**	19.4 ± 13.7	18.2 ± 12.7	0.772
NIHSS at 14 days, Mean±SD	17.5 ± 15.0	12.8 ± 12.7	0.133	17.5 ± 15.0	17.3 ± 13.9	0.912
Discharge mRS, Mean±SD	3.9 ± 1.5	3.6 ± 1.6	0.297	3.9 ± 1.5	4.1 ± 1.5	0.419
mRS 0–2 at 90 days, n(%)	15(41.7%)	96(43.6%)	0.968	15(41.7%)	23(31.9%)	0.433
Mortality, n(%)	6(16.7%)	31(14.1%)	0.879	6(16.7%)	12(16.7%)	1.000

Procedural time intervals—including onset-to-door time, onset-to-needle time (among patients receiving rt-PA), onset-to-puncture time, and onset-to-reperfusion time—were not significantly different between the two groups in the primary cohort. In the PSM cohort, door-to-reperfusion time was significantly shorter in BAO patients compared with ACS patients (3.2 ± 1.1 hours vs. 4.3 ± 2.9 hours, P = 0.007), while other time intervals remained comparable.

### Neurological outcomes

Outcome data are presented in Table 2 and S1 Fig. In the primary cohort, hemorrhagic transformation was significantly less frequent in BAO patients than in ACS patients (19.4% vs. 43.6%, P = 0.010). Parenchymal hemorrhage was observed in 11.1% of BAO patients and 20.9% of ACS patients, a difference that did not reach statistical significance (P = 0.255). In the PSM cohort, these findings were consistent: hemorrhagic transformation remained significantly lower in the BAO group (19.4% vs. 48.6%, P = 0.006), and parenchymal hemorrhage showed a trend toward being less frequent in BAO patients (11.1% vs. 26.4%, P = 0.083).

The mean NIHSS score at 24 hours was higher in BAO patients than in ACS patients in the primary cohort (19.4 ± 13.7 vs. 14.3 ± 11.1, P = 0.046), but this difference was no longer significant in the PSM cohort (19.4 ± 13.7 vs. 18.2 ± 12.7, P = 0.772). Similarly, 14-day NIHSS scores were comparable between the two groups in both the primary and PSM cohorts.

The distribution of mRS scores at 90 days is shown in S1 Fig. The rates of good functional outcome (mRS 0–2) at 90 days were similar between BAO and ACS patients in both the primary cohort (41.7% vs. 43.6%, P = 0.968) and the PSM cohort (41.7% vs. 31.9%, P = 0.433). The overall 90-day mortality rate was 14.5% (37/256) in the primary cohort. Ninety-day mortality was comparable between the BAO and ACS groups in both the primary cohort (16.7% vs. 14.1%, P = 0.879) and the PSM cohort (16.7% vs. 16.7%, P = 1.000).

### Predictors of poor outcome and mortality

Collinearity among predictor variables was assessed prior to regression modeling (S2 Fig). Univariate and multivariate logistic regression analyses for poor outcome (mRS 3–6) at 90 days and mortality in patients with LAO undergoing EVT are presented in Table 3.

**Table 3:** The multivariate logistic regression analysis of mRS 3-6 at 90 days and mortality.

Variable	mRS 3–6 at 90 days				Mortality			
	Univariate Odds ratio (95% CI)	*P-*value	Multivariate Odds Ratio (95% CI)	*P-*value	Univariate Odds ratio (95% CI)	*P-*value	Multivariate Odds Ratio (95% CI)	*P-*value
Location	0.923	0.825	–	–	0.820	0.684	**–**	**–**
BAO vs ACS	0.452-1.884				0.316-2.132			
Age	1.017	**0.059**	1.012	0.222	1.040	**0.010**	1.037	**0.020**
Per 1-year Increase	0.999-1.036		0.993-1.032		1.010-1.071		1.006-1.069	
Male	0.823	0.439	–	–	1.535	0.235	–	–
Yes vs No	0.501-1.350				0.756-3.116			
Hypertension	1.742	**0.036**	1.511	0.146	1.554	0.266	–	–
Yes vs No	1.037-2.925		0.866-2.636		0.715-3.376			
Diabetes Mellitus	1.732	0.120	–	–	2.088	**0.077**	2.025	0.107
Yes vs No	0.867-3.462				0.924-4.714		0.858-4.780	
AF History	1.140	0.619	–	–	1.257	0.529	–	–
Yes vs No	0.680-1.911				0.617-2.564			
Tobacco Use	0.677	0.195	–	**–**	0.789	0.597	**–**	**–**
Yes vs No	0.375-1.222				0.327-1.904			
Hypercholesterolemia	1.357	0.633	–	–	1.333	0.720	–	–
Yes vs No	0.387-4.756				0.276-6.431			
Baseline mRS	1.170	0.236	–	–	1.299	**0.053**	1.291	0.078
Per 1-point Increase	0.903-1.517				0.997-1.692		0.971-1.716	
Baseline NIHSS score	1.068	**<0.001**	1.052	**0.009**	1.074	**<0.001**	1.070	**0.002**
Per 1-point Increase	1.029-1.108		1.013-1.092		1.030-1.120		1.024-1.119	
Cardioembolic Etiology	1.380	0.330	–	–	0.592	0.217	–	–
Yes vs No	0.721-2.650				0.257-1.362			
Ischemic Core Volume	1.013	**0.010**	1.006	0.262	1.005	0.169	**–**	**–**
Per 1-mL Increase	1.003-1.022		0.996-1.016		0.998-1.013			
Hypoperfusion Volume	1.004	**0.090**	1.001	0.558	1.004	0.104	–	–
Per 1-mL Increase	0.999-1.008		0.997-1.006		0.999-1.008			
Mismatch Volume	1.001	0.776	–	**–**	1.003	0.293	**–**	**–**
Per 1-mL Increase	0.996-1.005				0.998-1.008			
Mismatch Ratio	0.858	**0.012**	–	**–**	0.996	0.955	**–**	**–**
Per 0.1-unit Increase	0.762-0.967				0.854-1.161			
ASPECT	0.747	**<0.001**	0.813	**0.021**	0.930	0.489	–	**–**
Per 1-point Increase	0.636-0.876		0.682-0.969		0.757-1.142			
Onset_door_time	1.032	0.186	–	**–**	0.986	0.687	–	**–**
Per 1-hour Increase	0.985-1.082				0.923-1.055			
Intravenous_rt_PA	1.427	0.268	–	**–**	1.561	0.275	–	**–**
Yes vs No	0.761-2.674				0.702-3.473			
Onset_puncture_time	1.023	0.308	–	**–**	0.982	0.567	–	**–**
Per 1-hour Increase	0.979-1.068				0.921-1.046			
Onset-Reperfusion Time	1.034	0.129	–	**–**	0.978	0.482	–	**–**
Per 1-hour Increase	0.990-1.078				0.920-1.040			
Door_reperfusion_time	1.045	0.412	–	**–**	0.927	0.405	–	**–**
Per 1-hour Increase	0.941-1.160				0.776-1.108			
Device	0.840	0.714	–	**–**	1.119	0.863	–	**–**
Stent+Asp vs Sten	0.329-2.142				0.310-4.049			
Device	0.611	0.272	–	**–**	0.569	0.460	–	**–**
Asp only vs Sten	0.254-1.471				0.127-2.541			

For poor functional outcome (mRS 3–6 at 90 days), univariate analysis identified the following variables as candidates (P < 0.1): age (OR 1.017; 95% CI, 0.999–1.036; P = 0.059), hypertension (OR 1.742; 95% CI, 1.037–2.925; P = 0.036), baseline NIHSS score (OR 1.068; 95% CI, 1.029–1.108; P < 0.001), ischemic core volume (OR 1.013; 95% CI, 1.003–1.022; P = 0.010), hypoperfusion volume (OR 1.004; 95% CI, 0.999–1.008; P = 0.090), and ASPECT (OR 0.747; 95% CI, 0.636–0.876; P < 0.001). Occlusion location (BAO vs. ACS) was not a significant predictor (OR 0.923; 95% CI, 0.452–1.884; P = 0.825). Although the mismatch ratio also reached significance in univariate analysis (OR 0.858 per 0.1-unit increase; 95% CI, 0.762–0.967; P = 0.012), it was excluded from the multivariate model owing to high collinearity with ischemic core volume and hypoperfusion volume, from which it is directly derived (S2 Fig). Multivariate logistic regression revealed that higher baseline NIHSS score (OR 1.052; 95% CI, 1.013–1.092; P = 0.009) and lower ASPECT (OR 0.813; 95% CI, 0.682–0.969; P = 0.021) were independent predictors of poor outcome.

For mortality, univariate analysis identified the following candidates (P < 0.1): age (OR 1.040; 95% CI, 1.010–1.071; P = 0.010), diabetes mellitus (OR 2.088; 95% CI, 0.924–4.714; P = 0.077), baseline mRS score (OR 1.299; 95% CI, 0.997–1.692; P = 0.053), and baseline NIHSS score (OR 1.074; 95% CI, 1.030–1.120; P < 0.001). Again, occlusion location was not a significant predictor (OR 0.820; 95% CI, 0.316–2.132; P = 0.684). In multivariate analysis, older age (OR 1.037; 95% CI, 1.006–1.069; P = 0.020) and higher baseline NIHSS score (OR 1.070; 95% CI, 1.024–1.119; P = 0.002) were independent predictors of mortality.

## Discussion

This study aimed to compare the clinical characteristics and outcomes of patients with acute BAO and anterior circulation stroke (ACS) treated with endovascular procedures based on CT perfusion. The results showed significant differences in the pathogenesis of stroke and treatment procedures between BAO and ACS, which influenced clinical outcomes.

In this study, patients with LVO were classified as having cardioembolic stroke and other determined etiologies. Compared with patients with ACS, patients with BAO had a lower rate of cardioembolic stroke. The anatomical structure of the carotid artery may provide better access for the thrombus to enter anterior circulation. Additionally, the average age was higher in ACS patients, consistent with the evidence that atrial fibrillation (AF) is a risk factor for cardioembolism [15]. Previous studies have shown that cardioembolism is less common in posterior circulation stroke (PCS) [16]. These findings suggest that the anatomical structures of the anterior and posterior circulations are unique and distinct, leading to different stroke pathogeneses.

Consistent with previous studies comparing ACS and PCS [17–19], patients with BAO had higher baseline NIHSS scores and larger ischemic volume. Higher baseline NIHSS scores is partly due to the limitations of the NIHSS in assessing neurological deficits in patients with PCS, as it is highly weighted toward deficits occurring in ACS, such as aphasia and hemiparesis, while neglecting deficits such as unsteady gait, dysphagia, or oculomotor disorders [20]. Larger ischemic volume is partly due to limited collateral circulation in posterior circulation brain tissue [18]. This is also the reason excluding baseline NIHSS from the PMS model.

The time from onset to door and onset to picture did not differ between the BAO and ACS patients. However, the mismatch volume/penumbra volume ratio was significantly larger in patients with BAO. Salvageable brain tissue in the posterior circulation might persist beyond the time window for ACS because of a higher proportion of white matter in the brainstem and better collateral arterial flow [21]. It has been shown that patients with PCS also benefit from mechanical thrombectomy when performed beyond 6 hours after symptom onset [22]. This study provides new neuroimaging evidence, suggesting that extended time window thrombectomy based on CT perfusion might be a future research direction for BAO.

Hemorrhagic complications, including hemorrhagic transformation (HT) and parenchymal hematoma (PH), are common in ACS patients. This may be explained by the larger volume of ischemic tissue in the anterior circulation [23]. Another reasonable explanation could be the higher hemodynamic flow in the carotid artery than in the basilar artery, leading to higher vascular bed pressure in the anterior circulation. It has been hypothesized that a smaller infarct volume in PCS patients contributes to a lower rate of hemorrhage, and pretreatment permeability abnormalities of the blood-brain barrier rarely occur in acute PCS [24]. This study reinforces the finding that acute revascularization therapy with mechanical thrombectomy can be safely performed in patients with acute BAO. Further analysis showed that HT and PH were significantly associated with poor outcomes and mortality in LAO patients.

The time from onset to door, onset to puncture, onset to reperfusion, EVT procedure, and recanalization rates were similar between the BAO and ACS patients. The 3-month poor outcome and mortality rates were also similar between the two groups. Studies that have specifically focused on BAO have reported poorer outcomes and mortality in patients with acute basilar occlusion treated with intravenous thrombolysis (IVT) or intra-arterial thrombolysis (IAT) owing to low recanalization rates [25,26]. The introduction of mechanical thrombectomy for the treatment of vertebrobasilar occlusion results in a decrease in mortality and an increase in the likelihood of poor outcomes at 90 days [27–29]. While CT perfusion mismatch is predominantly used to assess eligibility in patients with ACS, it is less reliable in PCS. This study showed that CT perfusion is reliable for assessing infarcts in the posterior fossa or brainstem of patients with acute BAO. The mismatch volume/penumbra volume was significantly larger in BAO than in ACS, while the onset-to-picture time was similar between the two groups. Further studies are needed to explore the therapeutic window of opportunity for reperfusion treatment of BAO.

Although this study found that the location of the stroke (ACS vs. BAO) was not an independent predictor of poor outcomes and mortality, several factors, including age and baseline neurological deficits, were identified to affect functional outcomes and mortality after mechanical thrombectomy. Older age predicted poor functional outcomes at 90 days. The NIHSS score was a significant predictor of both poor outcomes and mortality. The NIHSS has limitations in assessing common posterior circulation stroke symptoms (such as unsteady gait, dysphagia, and oculomotor disorders). The mRS score, although the standard functional endpoint in stroke trials, may incompletely capture clinically meaningful residual deficits frequently observed after posterior circulation stroke. Consequently, comparable mRS outcomes may underestimate differences in the actual burden of disability between posterior and anterior circulation stroke patients. This relationship was observed when ACS and PCS thrombectomies were combined. Larger multicenter data are required to further study this effect in patients with BAO.

### Limitations

This study has several important limitations. First, this was a single-center retrospective observational study, which may be subject to selection bias and unmeasured confounding. Second, the sample size of patients with BAO was relatively small compared with ACS group, which may limit the statistical power and generalizability of the results. Third, all patients included in this study underwent CTP imaging, and no control group treated without CTP guidance was available. Therefore, we cannot determine whether CTP-based selection improved patient eligibility, treatment decisions, or clinical outcomes compared with conventional non-perfusion imaging strategies. Fourth, despite the use of propensity score matching to balance baseline characteristics between groups, residual differences in baseline NIHSS scores persisted, which may introduce residual confounding that could not be fully adjusted for. This may affect the interpretation of the comparative effectiveness and safety of EVT between BAO and ACS patients. Finally, mRS as the primary functional outcome may incompletely capture posterior circulation–specific deficits such as dysphagia, ataxia, and dysarthria, which could lead to underestimation of the true disability burden in BAO patients.

These limitations should be considered when interpreting the comparative effectiveness and safety of EVT in patients with BAO versus ACS.

## Conclusion

This study suggests that EVT based on CT perfusion in BAO is associated with a lower risk of HT and similar effectiveness to ACS. Patients with BAO also seem to benefit from EVT when started within 24 h after symptom onset. The study demonstrated that patients are generally younger and had a lower rate of cardioembolism and HT. The most important factors determining the clinical outcome of patients with LAO are age and initial stroke severity.

## Supporting information

S1 Fig Outcomes of patients with CTP-guided EVT. (A) mRS score at 90 days of BAO patients and ACS patients. (B) Mortality of patients and ACS patients.
(TIF)

S2 Fig Collinearity among predictor variables. Collinearity among predictor variables.
(TIF)

S1 Table Clinical characteristics between patients with ICA and MCA. (DOCX)

S1 File Supporting Information. Research underlying data.
(XLSX)
