# Supplementary figures and images for: Perfusion imaging findings, and outcomes between computed tomography perfusion selected basilar artery occlusion and anterior circulation stroke patients undergoing endovascular treatment
Source: PLoS One. 2026 Jul 14;21(7):e0353204. doi: 10.1371/journal.pone.0353204 (PMC13367688; doi:10.1371/journal.pone.0353204)

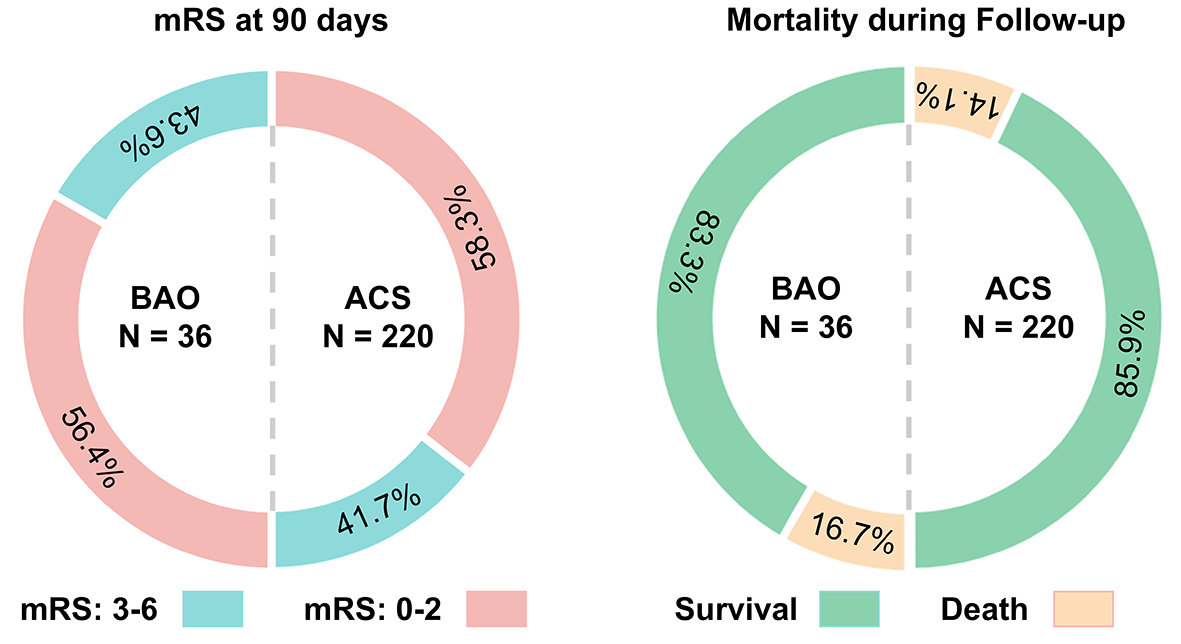

Supplement: S1 Fig — (A) mRS score at 90 days of BAO patients and ACS patients. (B) Mortality of patients and ACS patients. (TIF) [file pone.0353204.s001.tif]

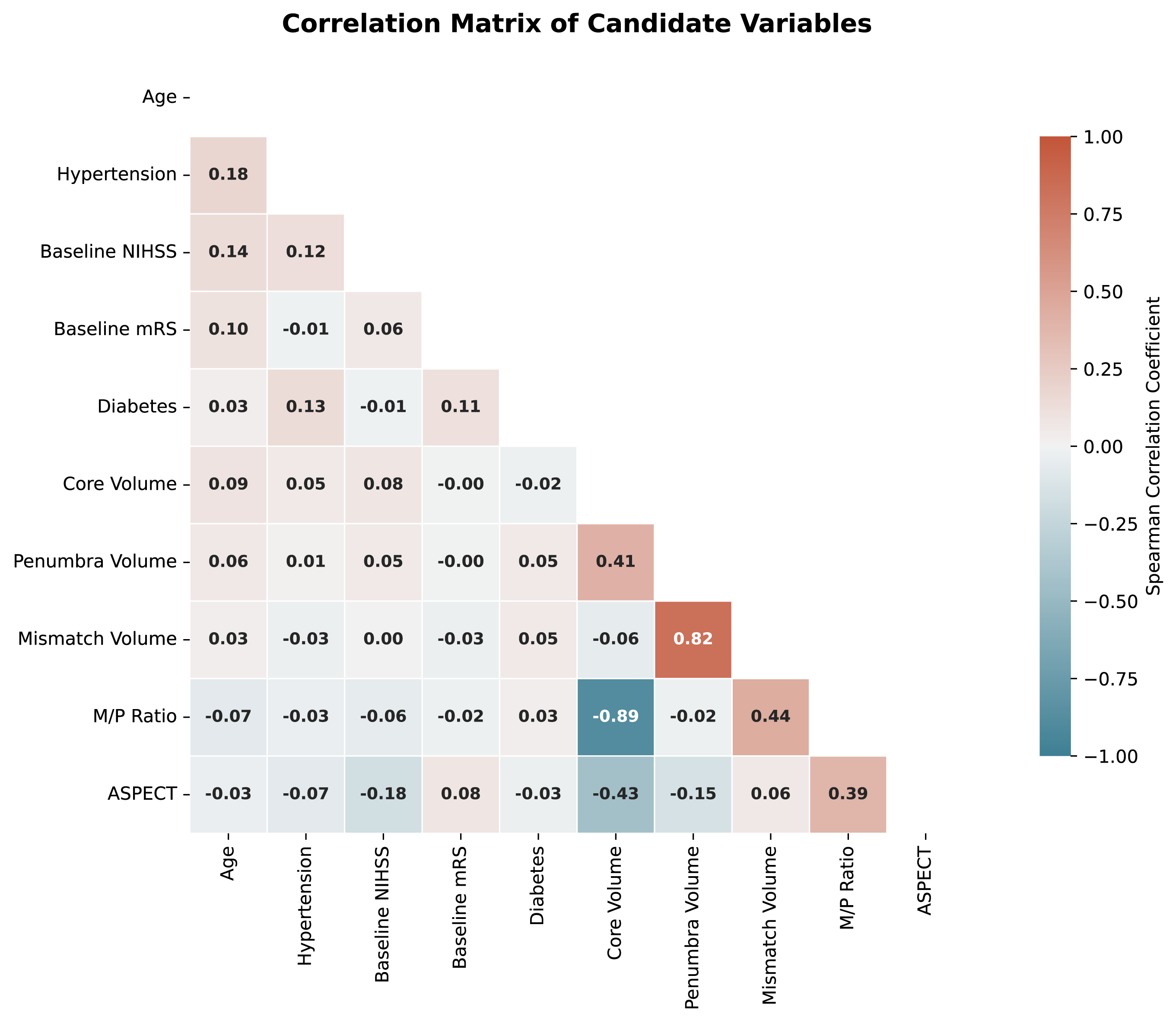

Supplement: S2 Fig — Collinearity among predictor variables. (TIF) [file pone.0353204.s002.tif]
