# Supplementary material for: Perfusion imaging findings, and outcomes between computed tomography perfusion selected basilar artery occlusion and anterior circulation stroke patients undergoing endovascular treatment
Source: PLoS One. 2026 Jul 14;21(7):e0353204. doi: 10.1371/journal.pone.0353204 (PMC13367688; doi:10.1371/journal.pone.0353204)
Supplement: S1 Table — (DOCX) [file pone.0353204.s003.docx]

**S1 Table.Clinical characteristics between patients with ICA and MCA.**

| **Variables** | ICA  (n=77) | MCA  (n=141) | *P* |
| --- | --- | --- | --- |
| Age, Mean ± SD | 68.3±13.7 | 68.9±14.3 | 0.789 |
| Sex |  |  | 0.258 |
| Male | 35 | 76 |  |
| Female | 42 | 65 |  |
| Hypertension |  |  | 0.656 |
| Yes | 52 | 90 |  |
| No | 25 | 51 |  |
| Diabetes mellitus |  |  | 0.846 |
| Yes | 11 | 22 |  |
| No | 66 | 119 |  |
| AF history |  |  | 0.884 |
| Yes | 28 | 54 |  |
| No | 49 | 87 |  |
| Tobacco use |  |  | 0.616 |
| Yes | 16 | 35 |  |
| No | 61 | 106 |  |
| Hypercholesterolemia |  |  | >0.999 |
| Yes | 2 | 4 |  |
| No | 75 | 137 |  |
| Baseline NIHSS Score,  Mean ± SD | 15.3±6.4 | 14.9±6.7 | 0.581 |
| Baseline mRS Score,  Mean ± SD | 0.3±0.9 | 0.4±1.0 | 0.193 |
| APECT, Mean ± SD | 7.8±1.5 | 7.9±1.7 | 0.768 |
| Stroke etiology |  |  | 0.345 |
| Cardioembolic | 61 | 120 |  |
| Others | 16 | 21 |  |
| Ischemic Core Volume,  Mean ± SD | 33.7±37.6 | 34.2±35.8 | 0.209 |
| Hypoperfusion Volume, Mean ± SD | 124.9±69.4 | 119.3±72.3 | 0.253 |
| Mismatch Volume,  Mean ± SD | 85.6±59.5 | 88.3±61.7 | 0.457 |
| Mismatch ratio,  Mean ± SD | 0.8±0.3 | 0.7±0.2 | 0.963 |
| Treatment Method,n(%) |  |  |  |
| Intravenous rt-PA | 15(19.5%) | 30(21.3%) | 0.491 |
| Device Use |  |  |  |
| Stent retriever | 63(81.8%) | 125(88.6%) |  |
| Stent + Aspiration | 4(5.1%) | 8(5.6%) |  |
| Aspiration only | 6(7.8%) | 18(8.5%) |  |
| Treatment Times, Mean ± SD |  |  |  |
| Onset-Door Time | 6.1±6.3 | 6.7±6.0 | 0.299 |
| Onset-Needle Time(rt-PA Only) | 3.2±4.9 | 3.7±5.8 | 0.386 |
| Onset-Puncture Time | 8.4±5.5 | 9.1±6.1 | 0.477 |
| Onset-Reperfusion Time | 10.0±5.9 | 10.3±6.3 | 0.707 |
| Door-Reperfusion Time | 4.1±3.0 | 3.6±2.1 | 0.239 |
| Complications, n (%) |  |  |  |
| Hemorrhagic Transformation | 32(41.5%) | 63(44.7%) | 0.671 |
| Parenchymal Hemorrhage | 15(19.5%) | 30(21.3%) | 0.862 |
| Clinical Outcomes |  |  |  |
| NIHSS at 24h , Mean±SD | 16.1±10.8 | 15.2±11.4 | 0.548 |
| NIHSS at 14 days , Mean±SD | 14.3±12.7 | 13.5±12.8 | 0.671 |
| Discharge mRS, Mean±SD | 3.7±1.2 | 3.5±1.6 | 0.249 |
| mRS 0-2 at 90 days, n(%) | 32(41.6%) | 63(44.7%) | 0.671 |
| Mortality, n(%) | 11(14.2%) | 19(13.5%) | 0.841 |
