# Supplementary material for: Perfusion imaging findings, and outcomes between computed tomography perfusion selected basilar artery occlusion and anterior circulation stroke patients undergoing endovascular treatment
Source: PLoS One. 2026 Jul 14;21(7):e0353204. doi: 10.1371/journal.pone.0353204 (PMC13367688; doi:10.1371/journal.pone.0353204)
Supplement: Attachment — Submitted filename: Response to reviwer 2.doc [file pone.0353204.s006.doc]

Dear reviewer 2:

Please find our revision for the above reference manuscript entitled: “Endovascular treatment based on computer tomography perfusion for basilar artery occlusion compared with anterior circulation stroke” for consideration for publishing in PLOS ONE as an original article.

We have revised our manuscript based upon the comments from the reviewer. We would like to take this opportunity to thank you and the reviewers for taking the time to review our submission, and for your thoughtful comments and suggestions to improve the quality of our manuscript.

We provide a point-by point response to the reviewers’ comments below.

This study, based on a case-control study, compared the outcomes of endovascular therapy (EVT) in patients with vertebrobasilar artery occlusion (BAO) versus those with anterior circulation stroke (ACS) using computed tomography perfusion (CTP). The results indicate that the efficacy of EVT in CTP-selected BAO patients is similar to that in ACS patients. BAO patients can also benefit from EVT initiated within 24 hours of symptom onset.

1.Did this study exclude patients with tandem lesions? If not, were subgroup analyses or sensitivity analyses performed for these patients to control for confounding effects?

**Response:**

Thank you for your question. There are only two tandem lesions patients in BAO group which combined with vertebral artery occlusion. All patients including BAO and ACS were reperfusion. So Our study did not exclude the patients with tandem lesions.

2.In the assessment based on computed tomography perfusion (CTP), were quantitative parameters such as cerebral blood flow (CBF) and cerebral blood volume (CBV) specifically included? Can these parameters be used for patient selection or as prognostic predictors?

**Response:**

Thank you for your question. These parameters were including to evaluate patients before EVT. The hypoperfusion volume was defined using a delay time (DT) threshold of >3 seconds. The ischemic core volume was calculated using a relative cerebral blood flow (rCBF) threshold of <30%.(Described in paragraph “**Imaging protocol**”). However we did not use these to patient selection or as prognostic predictors delectly. Maybe we could study the relationships between these quantitative parameters and clinical outcomes retrospectively.

3. Given that the time of admission or imaging detection for patients with posterior circulation infarction is often later than for those with anterior circulation occlusion, I would suggest that the authors perform a stratified analysis based on the time from symptom onset to randomization (or to EVT initiation) to better assess whether the treatment effect differs across different time windows.

**Response:**

Thank you for your suggestion. In our study, ‘two groups’ time of onset-door and onset-puncture for patients are similar (Table 2). So we did not perform a stratified analysis based on the time from symptom onset to randomization.
