# Supplementary material for: Perfusion imaging findings, and outcomes between computed tomography perfusion selected basilar artery occlusion and anterior circulation stroke patients undergoing endovascular treatment
Source: PLoS One. 2026 Jul 14;21(7):e0353204. doi: 10.1371/journal.pone.0353204 (PMC13367688; doi:10.1371/journal.pone.0353204)
Supplement: Attachment — Submitted filename: revised to editor R2.docx [file pone.0353204.s007.docx]

Dear editor:

Please find our revision for the above reference manuscript entitled: “Endovascular treatment based on computer tomography perfusion for basilar artery occlusion compared with anterior circulation stroke” for consideration for publishing in PLOS ONE as an original article.

We have revised our manuscript based upon the comments from the editor. We would like to take this opportunity to thank you and the reviewers for taking the time to review our submission, and for your thoughtful comments and suggestions to improve the quality of our manuscript.

We provide a point-by point response to the reviewers’ comments below.

1. Study framing and title
The current title and several statements throughout the Abstract, Discussion, and Conclusions imply that the study evaluates the efficacy of CT perfusion-guided endovascular treatment. However, all included patients underwent CT perfusion imaging and no comparator group without perfusion imaging is available. Consequently, the study cannot determine whether CT perfusion guidance improves patient selection or clinical outcomes. The title and corresponding statements should therefore be revised to more accurately reflect the study design, namely a comparison of clinical characteristics, perfusion imaging findings, and outcomes between CTP-selected basilar artery occlusion and anterior circulation stroke patients undergoing EVT, rather than an evaluation of the effectiveness of a CTP-guided strategy.
**Response:**

**Thanks for your suggestion. We have rewritten the title as “Perfusion imaging findings, and outcomes between CTP-selected basilar artery occlusion and anterior circulation stroke patients undergoing EVT”.**
2. Introduction and Discussion
The statement in the Introduction:
“Due to limited scientific evidence, the approaches to EVT for BAO remain heterogeneous due to the lack of data from randomized clinical trials.” should be removed or substantially revised, as it is no longer supported by the available evidence.
Similarly, the opening sentence of the Discussion: “Despite recent breakthroughs in the treatment of ischemic stroke caused by large artery occlusion (LAO), the optimal treatment for basilar artery occlusion (BAO) remains unclear, as BAO patients were excluded from recent randomized controlled trials.” should be removed, as it is factually incorrect. The ATTENTION and BAOCHE trials, together with BASICS and BEST, have established the benefit of EVT in appropriately selected BAO patients. The discussion should instead focus on the remaining uncertainties regarding imaging selection, perfusion assessment, and specific clinical subgroups.
**Thanks for your correction. We have deleted the sentence in revised manuscript.**

3. Propensity score matching and statistical methods
   The propensity score matching methodology should be incorporated into the Statistical Analysis section to improve the organization and transparency of the Methods.
   Moreover, the manuscript describes the matched cohort as "well balanced," despite a substantial residual difference in baseline NIHSS score. Given that NIHSS is one of the strongest determinants of functional outcome and mortality, this imbalance deserves explicit discussion. Importantly, the known limitations of the NIHSS in posterior circulation stroke do not diminish the significance of this finding; rather, the persistence of a significantly higher NIHSS score in the BAO cohort despite the underrepresentation of posterior circulation symptoms suggests that these patients may have had an even greater neurological severity than reflected by the scale. The authors should clarify the rationale for excluding baseline NIHSS from the propensity score model, discuss the potential impact of this residual imbalance, and reconsider describing the matched cohort as "well balanced."

**According to your request, the PSM in method section has been incorporated into the Statistical Analysis section.**

**The paragraph in the discussion “Consistent with previous studies comparing ACS and PCS [17-19], patients with BAO had higher baseline NIHSS scores and larger ischemic volume. Higher baseline NIHSS scores is partly due to the limitations of the NIHSS in assessing neurological deficits in patients with PCS, as it is highly weighted toward deficits occurring in ACS, such as aphasia and hemiparesis, while neglecting deficits such as unsteady gait, dysphagia, or oculomotor disorders[20]. Larger ischemic volume is partly due to limited collateral circulation in posterior circulation brain tissue[18].” explains why BAO has a high NIHSS score, which is a disease characteristic that cannot be well balanced, otherwise it would be difficult to include matched patients. The imbalance of this baseline feature and the relative balance of the results indicate that BAO may have gained greater benefits after this treatment.**

4. Outcome assessment
The Discussion should acknowledge that the modified Rankin Scale, although the standard functional endpoint in stroke trials, may incompletely capture clinically meaningful residual deficits frequently observed after posterior circulation stroke. Consequently, comparable mRS outcomes may underestimate differences in the actual burden of disability between posterior and anterior circulation stroke patients.
**Thanks for your correction. We have added the sentence “the mRS score, although the standard functional endpoint in stroke trials, may incompletely capture clinically meaningful residual deficits frequently observed after posterior circulation stroke. Consequently, comparable mRS outcomes may underestimate differences in the actual burden of disability between posterior and anterior circulation stroke patients.” in revised manuscript.**

5. Applicability of CT perfusion parameters
Given the unique anatomical and hemodynamic characteristics of the posterior circulation, the Discussion would benefit from a brief consideration of the applicability and current evidence supporting conventional CT perfusion thresholds in basilar artery occlusion. The authors should clarify that the present study applies established perfusion parameters but does not aim to validate or optimize CTP thresholds for posterior circulation stroke.
**Thanks for your correction. We have added the sentence “the present study applies established perfusion parameters but does not aim to validate or optimize CTP thresholds for posterior circulation stroke.” in revised manuscript.**

“
6. Limitations
The current Limitations section is overly concise and should be substantially expanded. In addition to the retrospective design and the relatively small BAO cohort, the authors should explicitly discuss the absence of a non-CTP comparator group, the potential for residual confounding related to the persistence of baseline NIHSS differences after propensity score matching, and the implications of these limitations when interpreting the comparative effectiveness and safety of EVT between BAO and ACS.
**Thanks for your correction. We have** **rewritten the limitation as** **“This study has several important limitations. First, this was a single-center retrospective observational study, which may be subject to selection bias and unmeasured confounding. Second, the sample size of patients with BAO was relatively small compared with ACS group, which may limit the statistical power and generalizability of the results. Third, all patients included in this study underwent CTP imaging, and no control group treated without CTP guidance was available. Therefore, we cannot determine whether CTP-based selection improved patient eligibility, treatment decisions, or clinical outcomes compared with conventional non-perfusion imaging strategies. Fourth, despite the use of propensity score matching to balance baseline characteristics between groups, residual differences in baseline NIHSS scores persisted, which may introduce residual confounding that could not be fully adjusted for. This may affect the interpretation of the comparative effectiveness and safety of EVT between BAO and ACS patients. Finally, mRS as the primary functional outcome may incompletely capture posterior circulation–specific deficits such as dysphagia, ataxia, and dysarthria, which could lead to underestimation of the true disability burden in BAO patients.**

**These limitations should be considered when interpreting the comparative effectiveness and safety of EVT in patients with BAO versus ACS.”**

7. Manuscript presentation
The overall presentation of the manuscript requires careful revision. The current version contains numerous formatting inconsistencies, including irregular punctuation and spacing, figure legends embedded within the main text, and other editorial artifacts that adversely affect readability. The manuscript should be carefully proofread to ensure that the text, figures, legends, and tables are presented in a clear and consistent manner.
**Thanks for your correction. We have checked numerous formatting inconsistencies, including irregular punctuation and spacing, figure legends embedded in revised manuscript.**
