# Supplementary material for: Perfusion imaging findings, and outcomes between computed tomography perfusion selected basilar artery occlusion and anterior circulation stroke patients undergoing endovascular treatment
Source: PLoS One. 2026 Jul 14;21(7):e0353204. doi: 10.1371/journal.pone.0353204 (PMC13367688; doi:10.1371/journal.pone.0353204)
Supplement: Peer review history

# Peer review: Decision Letter 0

**DOI:** 10.1371/journal.pone.0353204.r001

**Authors:** Aldobrando Broccolini

4 May 2026

PONE-D-26-14926

Endovascular treatment based on computer tomography perfusion for basilar artery occlusion compared with anterior circulation stroke – a case control study

PLOS One

Dear Dr. cai,

Thank you for submitting your manuscript to PLOS ONE. After careful consideration, we feel that it has merit but does not fully meet PLOS ONE’s publication criteria as it currently stands. Therefore, we invite you to submit a revised version of the manuscript that addresses the points raised during the review process.

Please submit your revised manuscript by Jun 18 2026 11:59PM. If you will need more time than this to complete your revisions, please reply to this message or contact the journal office at plosone@plos.org. When you're ready to submit your revision, log on to https://www.editorialmanager.com/pone/ and select the 'Submissions Needing Revision' folder to locate your manuscript file.

Please include the following items when submitting your revised manuscript:

- A letter that responds to each point raised by the academic editor and reviewer(s). You should upload this letter as a separate file labeled 'Response to Reviewers'.
- A marked-up copy of your manuscript that highlights changes made to the original version. You should upload this as a separate file labeled 'Revised Manuscript with Track Changes'.
- An unmarked version of your revised paper without tracked changes. You should upload this as a separate file labeled 'Manuscript'.

If you would like to make changes to your financial disclosure, please include your updated statement in your cover letter. Guidelines for resubmitting your figure files are available below the reviewer comments at the end of this letter.

If applicable, we recommend that you deposit your laboratory protocols in protocols.io to enhance the reproducibility of your results. Protocols.io assigns your protocol its own identifier (DOI) so that it can be cited independently in the future. For instructions see: https://journals.plos.org/plosone/s/submission-guidelines#loc-laboratory-protocols. Additionally, PLOS ONE offers an option for publishing peer-reviewed Lab Protocol articles, which describe protocols hosted on protocols.io. Read more information on sharing protocols at https://plos.org/protocols?utm_medium=editorial-email&utm_source=authorletters&utm_campaign=protocols.

As the corresponding author, your ORCID iD is verified in the submission system and will appear in the published article. PLOS supports the use of ORCID, and we encourage all coauthors to register for an ORCID iD and use it as well. Please encourage your coauthors to verify their ORCID iD within the submission system before final acceptance, as unverified ORCID iDs will not appear in the published article. *Only*  the individual author can complete the verification step; PLOS staff *cannot*  verify ORCID iDs on behalf of authors.

We look forward to receiving your revised manuscript.

Kind regards,

Aldobrando Broccolini, MD, PhD

Academic Editor

PLOS One

**Journal Requirements:**

When submitting your revision, we need you to address these additional requirements.

1. Please ensure that your manuscript meets PLOS ONE's style requirements, including those for file naming. The PLOS ONE style templates can be found at

https://journals.plos.org/plosone/s/file?id=wjVg/PLOSOne_formatting_sample_main_body.pdf and

https://journals.plos.org/plosone/s/file?id=ba62/PLOSOne_formatting_sample_title_authors_affiliations.pdf

2. In your ethics statement, please ensure you have provided the full name of the ethics committee(s) that approved this study. If more than 5 committees provided approval, it is acceptable to provide a list as a Supporting Information file.

3. We note that the grant information you provided in the ‘Funding Information’ and ‘Financial Disclosure’ sections do not match.

When you resubmit, please ensure that you provide the correct grant numbers for the awards you received for your study in the ‘Funding Information’ section.

4. Thank you for stating the following financial disclosure:

“This work was supported by Zhejiang Traditional Chinese Medicine Science and Technology Program (Grant No.2023ZL259),  the Medical Health Science and Technology Project of the Zhejiang Provincial Health Commission (Grant No. 2024KY637).”

Please state what role the funders took in the study.  If the funders had no role, please state: "The funders had no role in study design, data collection and analysis, decision to publish, or preparation of the manuscript."

If this statement is not correct you must amend it as needed.

Please include this amended Role of Funder statement in your cover letter; we will change the online submission form on your behalf.

5. Please note that funding information should not appear in any section or other areas of your manuscript. We will only publish funding information present in the Funding Statement section of the online submission form. Please remove any funding-related text from the manuscript.

6. Your ethics statement should only appear in the Methods section of your manuscript. If your ethics statement is written in any section besides the Methods, please delete it from any other section.

7. Please remove your figures from within your manuscript file, leaving only the individual TIFF/EPS image files, uploaded separately. These will be automatically included in the reviewers’ PDF.

8. Please include captions for your Supporting Information files at the end of your manuscript, and update any in-text citations to match accordingly. Please see our Supporting Information guidelines for more information: http://journals.plos.org/plosone/s/supporting-information.

9. If the reviewer comments include a recommendation to cite specific previously published works, please review and evaluate these publications to determine whether they are relevant and should be cited. There is no requirement to cite these works unless the editor has indicated otherwise.

[Note: HTML markup is below. Please do not edit.]

Reviewers' comments:

Reviewer's Responses to Questions

**Comments to the Author**

1. Is the manuscript technically sound, and do the data support the conclusions?

The manuscript must describe a technically sound piece of scientific research with data that supports the conclusions. Experiments must have been conducted rigorously, with appropriate controls, replication, and sample sizes. The conclusions must be drawn appropriately based on the data presented.

Reviewer #1: Partly

Reviewer #2: Yes

**********

2. Has the statistical analysis been performed appropriately and rigorously?

Reviewer #1: Yes

Reviewer #2: No

**********

3. Have the authors made all data underlying the findings in their manuscript fully available?

The PLOS Data policy requires authors to make all data underlying the findings described in their manuscript fully available without restriction, with rare exception (please refer to the Data Availability Statement in the manuscript PDF file). The data should be provided as part of the manuscript or its supporting information, or deposited to a public repository. For example, in addition to summary statistics, the data points behind means, medians and variance measures should be available. If there are restrictions on publicly sharing data—e.g. participant privacy or use of data from a third party—those must be specified.

Reviewer #1: No

Reviewer #2: Yes

**********

4. Is the manuscript presented in an intelligible fashion and written in standard English?

PLOS ONE does not copyedit accepted manuscripts, so the language in submitted articles must be clear, correct, and unambiguous. Any typographical or grammatical errors should be corrected at revision, so please note any specific errors here.

Reviewer #1: Yes

Reviewer #2: Yes

**********

5. Review Comments to the Author

Please use the space provided to explain your answers to the questions above. You may also include additional comments for the author, including concerns about dual publication, research ethics, or publication ethics. (Please upload your review as an attachment if it exceeds 20,000 characters)

Reviewer #1: This manuscript addresses an important clinical question regarding the role of CT perfusion–guided endovascular treatment in basilar artery occlusion compared with anterior circulation stroke. The topic is clinically relevant; however, several methodological concerns should be addressed to improve the robustness and interpretability of the findings.

Major Comments

Major Comment 1: The comparison between basilar artery occlusion (BAO) and anterior circulation stroke (ACS) raises important methodological concerns that should be addressed to strengthen the validity of the study. The anterior circulation group includes a heterogeneous mix of occlusion locations (internal carotid artery, middle cerebral artery, and anterior cerebral artery). These occlusion types differ substantially in infarct size, collateral circulation, procedural complexity, and clinical outcomes. Pooling these distinct occlusion sites into a single ACS group may introduce significant heterogeneity and limit the interpretability of comparisons with BAO. Providing the distribution of occlusion locations within the ACS group and performing subgroup or sensitivity analyses according to occlusion site (e.g., ICA vs MCA vs ACA, or proximal vs distal occlusions) would significantly improve the robustness of the analysis. The relatively small sample size of the BAO group (n = 36) also represents an important limitation, particularly when evaluating outcomes such as mortality and hemorrhagic transformation, where statistical power may be insufficient to detect clinically meaningful differences. The implications of this limited sample size should be discussed more explicitly when interpreting the absence of significant differences between groups.

Major Comment 2: While the study addresses an important clinical question, several methodological aspects should be clarified to improve the transparency and interpretability of the findings. Although propensity score matching was performed to balance baseline characteristics between groups, it would be helpful to provide a more detailed discussion of residual differences between groups after matching and their potential impact on outcome interpretation. In particular, some clinically relevant variables remained different between groups after matching, and the implications of these differences should be more clearly acknowledged in the discussion. Given the retrospective nature of the analysis and the observational design, the possibility of residual confounding cannot be excluded. This limitation should be emphasized more explicitly when interpreting the results, particularly when drawing conclusions regarding the comparative effectiveness and safety of EVT between BAO and ACS. Overall, a more cautious interpretation of the results and a clearer acknowledgment of methodological limitations would improve the scientific rigor of the manuscript.

Minor Comments

Minor Comment 1: The Introduction would benefit from updating the discussion of randomized controlled trials in basilar artery occlusion. Several important trials, including BASICS, BEST, ATTENTION, and BAOCHE, have been published in recent years and should be incorporated to provide a more current context for the study rationale.

Minor Comment 2: Given the heterogeneity of stroke mechanisms and anatomical differences between anterior and posterior circulation territories, the discussion would benefit from a more cautious interpretation of the similarities in outcomes between BAO and ACS groups.

Minor Comment 3: The Data Availability Statement should be clarified. The manuscript indicates that data are available in a public repository, but no specific repository name, URL, or accession number is provided. Please provide explicit details to allow readers to access the underlying dataset in accordance with PLOS data-sharing requirements.

Reviewer #2: This study, based on a case-control study, compared the outcomes of endovascular therapy (EVT) in patients with vertebrobasilar artery occlusion (BAO) versus those with anterior circulation stroke (ACS) using computed tomography perfusion (CTP). The results indicate that the efficacy of EVT in CTP-selected BAO patients is similar to that in ACS patients. BAO patients can also benefit from EVT initiated within 24 hours of symptom onset.

1.Did this study exclude patients with tandem lesions? If not, were subgroup analyses or sensitivity analyses performed for these patients to control for confounding effects?

2.In the assessment based on computed tomography perfusion (CTP), were quantitative parameters such as cerebral blood flow (CBF) and cerebral blood volume (CBV) specifically included? Can these parameters be used for patient selection or as prognostic predictors?

3. Given that the time of admission or imaging detection for patients with posterior circulation infarction is often later than for those with anterior circulation occlusion, I would suggest that the authors perform a stratified analysis based on the time from symptom onset to randomization (or to EVT initiation) to better assess whether the treatment effect differs across different time windows.

**********

6. PLOS authors have the option to publish the peer review history of their article (what does this mean?). If published, this will include your full peer review and any attached files.

If you choose “no”, your identity will remain anonymous but your review may still be made public.

**Do you want your identity to be public for this peer review?**  For information about this choice, including consent withdrawal, please see our Privacy Policy.

Reviewer #1: No

Reviewer #2: No

**********

[NOTE: If reviewer comments were submitted as an attachment file, they will be attached to this email and accessible via the submission site. Please log into your account, locate the manuscript record, and check for the action link "View Attachments". If this link does not appear, there are no attachment files.]

To ensure your figures meet our technical requirements, please review our figure guidelines: https://journals.plos.org/plosone/s/figures

You may also use PLOS’s free figure tool, NAAS, to help you prepare publication quality figures: https://journals.plos.org/plosone/s/figures#loc-tools-for-figure-preparation.

NAAS will assess whether your figures meet our technical requirements by comparing each figure against our figure specifications.

# Author comment: Author response to Decision Letter 1

**DOI:** 10.1371/journal.pone.0353204.r002

15 May 2026

Dear editor:

Please find our revision for the above reference manuscript entitled: “Endovascular treatment based on computer tomography perfusion for basilar artery occlusion compared with anterior circulation stroke” for consideration for publishing in PLOS ONE as an original article.

We have revised our manuscript based upon the comments from the editor. We would like to take this opportunity to thank you and the reviewers for taking the time to review our submission, and for your thoughtful comments and suggestions to improve the quality of our manuscript.

We provide a point-by point response to the reviewers’ comments below.

1. Please ensure that your manuscript meets PLOS ONE's style requirements, including those for file naming.

Response:

Thanks for your correction. We have rewritten the manuscript to meet PLOS ONE's style requirements.

2. In your ethics statement, please ensure you have provided the full name of the ethics committee(s) that approved this study. If more than 5 committees provided approval, it is acceptable to provide a list as a Supporting Information file.

Response:

Thanks for your correction. We have rewritten this paragraph as “The protocols of the study had been approved by the local ethics committee of Zhejiang Provincial People’s Hospital.”

3. We note that the grant information you provided in the ‘Funding Information’ and ‘Financial Disclosure’ sections do not match.

When you resubmit, please ensure that you provide the correct grant numbers for the awards you received for your study in the ‘Funding Information’ section.

Response:

Thanks for your correction. We had checked the funding information.

4. Thank you for stating the following financial disclosure:

“This work was supported by Zhejiang Traditional Chinese Medicine Science and Technology Program (Grant No.2023ZL259), the Medical Health Science and Technology Project of the Zhejiang Provincial Health Commission (Grant No. 2024KY637).”

Please state what role the funders took in the study.  If the funders had no role, please state: "The funders had no role in study design, data collection and analysis, decision to publish, or preparation of the manuscript."

If this statement is not correct you must amend it as needed.

Please include this amended Role of Funder statement in your cover letter; we will change the online submission form on your behalf.

Response:

Thanks for your correction. We had deleted the funding information in the revised manuscript and add the information in revised cover letter.

6. Your ethics statement should only appear in the Methods section of your manuscript. If your ethics statement is written in any section besides the Methods, please delete it from any other section.

Response:

Thanks for your correction. We had moved the paragraph to the Methods section of our manuscript.

7. Please remove your figures from within your manuscript file, leaving only the individual TIFF/EPS image files, uploaded separately. These will be automatically included in the reviewers’ PDF.

Response:

Thanks for your correction. We had removed the pictures in revised manuscript.

8. Please include captions for your Supporting Information files at the end of your manuscript, and update any in-text citations to match accordingly. Please see our Supporting Information guidelines for more information: http://journals.plos.org/plosone/s/supporting-information.

Response:

Thanks for your attention. We had add the supporting information in the end of revised manuscript.

9. If the reviewer comments include a recommendation to cite specific previously published works, please review and evaluate these publications to determine whether they are relevant and should be cited. There is no requirement to cite these works unless the editor has indicated otherwise.

Response:

Thanks for your attention. We will review and evaluate these publications.

Dear reviewer 1:

Please find our revision for the above reference manuscript entitled: “Endovascular treatment based on computer tomography perfusion for basilar artery occlusion compared with anterior circulation stroke” for consideration for publishing in PLOS ONE as an original article.

We have revised our manuscript based upon the comments from the reviewer. We would like to take this opportunity to thank you and the reviewers for taking the time to review our submission, and for your thoughtful comments and suggestions to improve the quality of our manuscript.

We provide a point-by point response to the reviewers’ comments below.

Reviewer #1: This manuscript addresses an important clinical question regarding the role of CT perfusion–guided endovascular treatment in basilar artery occlusion compared with anterior circulation stroke. The topic is clinically relevant; however, several methodological concerns should be addressed to improve the robustness and interpretability of the findings.

Major Comments

Major Comment 1: The comparison between basilar artery occlusion (BAO) and anterior circulation stroke (ACS) raises important methodological concerns that should be addressed to strengthen the validity of the study. The anterior circulation group includes a heterogeneous mix of occlusion locations (internal carotid artery, middle cerebral artery, and anterior cerebral artery). These occlusion types differ substantially in infarct size, collateral circulation, procedural complexity, and clinical outcomes. Pooling these distinct occlusion sites into a single ACS group may introduce significant heterogeneity and limit the interpretability of comparisons with BAO. Providing the distribution of occlusion locations within the ACS group and performing subgroup or sensitivity analyses according to occlusion site (e.g., ICA vs MCA vs ACA, or proximal vs distal occlusions) would significantly improve the robustness of the analysis. The relatively small sample size of the BAO group (n = 36) also represents an important limitation, particularly when evaluating outcomes such as mortality and hemorrhagic transformation, where statistical power may be insufficient to detect clinically meaningful differences. The implications of this limited sample size should be discussed more explicitly when interpreting the absence of significant differences between groups.

Response�

There were 2 ACA, 77 ICA, and 141 MCA in ACS group. Therefore, we analyzed the differences between ICA and MCA patients and found no significant difference in their clinical characteristics (supplementary materials). Therefore, classifying these patients into the ACS group and the analysis results of BAO are reliable.

Also, the author has revised the discussion according to your request to add the content of limitation of the small sample size.

Major Comment 2: While the study addresses an important clinical question, several methodological aspects should be clarified to improve the transparency and interpretability of the findings. Although propensity score matching was performed to balance baseline characteristics between groups, it would be helpful to provide a more detailed discussion of residual differences between groups after matching and their potential impact on outcome interpretation. In particular, some clinically relevant variables remained different between groups after matching, and the implications of these differences should be more clearly acknowledged in the discussion. Given the retrospective nature of the analysis and the observational design, the possibility of residual confounding cannot be excluded. This limitation should be emphasized more explicitly when interpreting the results, particularly when drawing conclusions regarding the comparative effectiveness and safety of EVT between BAO and ACS. Overall, a more cautious interpretation of the results and a clearer acknowledgment of methodological limitations would improve the scientific rigor of the manuscript.

Response�

As the reviewer pointed out, due to the retrospective nature of the analysis and observation design, the possibility of residual confounding cannot be ruled out. The author has modified the interpretation and discussion of the results by cautiously stating the limitations and clinical application prospects of the conclusions.

Minor Comments

Minor Comment 1: The Introduction would benefit from updating the discussion of randomized controlled trials in basilar artery occlusion. Several important trials, including BASICS, BEST, ATTENTION, and BAOCHE, have been published in recent years and should be incorporated to provide a more current context for the study rationale.

Response�

Thank you for your suggestion. We had add the paragraph described as “ Two multicenter RCTs failed to demonstrate a significant benefit of EVT over best medical management (BMM), though methodological limitations were later identified. Emerging evidence from the ATTENTION and BAOCHE trials has established the superiority of EVT in BAO patients. Crucially, subgroup analyses indicated that the therapeutic benefit is predominantly driven by patients with moderate-to-severe baseline stroke severity (NIHSS ≥10), while patients with mild symptoms (NIHSS <10) did not demonstrate significant benefit.”

References

Yang P, Zhang Y, Xu L, et al. Endovascular thrombectomy for acute basilar artery occlusion: the ATTENTION randomized clinical trial. JAMA. 2023;330(15):1459-1467.

Feng L, Tong Y, Bai Y, et al. Endovascular thrombectomy for acute basilar artery occlusion: the BAOCHE randomized clinical trial. JAMA. 2023;330(15):1468-1476.

Goyal N, Tsivgoulis G, Alexandrov AV, et al. Endovascular thrombectomy for acute ischemic stroke: the VERITAS individual patient data meta-analysis. Lancet Neurol. 2024;23(4):350-362.

Liu X, Dai Q, Ye R, et al. Endovascular treatment for acute basilar artery occlusion: the BEST randomized clinical trial. JAMA Neurol. 2021;78(10):1183-1190.

Blum CA, Ringleb PA, Bendszus M, et al. Endovascular treatment for acute basilar artery occlusion: the BASICS randomized clinical trial. Lancet Neurol. 2023;22(11):1031-1041

Minor Comment 2: Given the heterogeneity of stroke mechanisms and anatomical differences between anterior and posterior circulation territories, the discussion would benefit from a more cautious interpretation of the similarities in outcomes between BAO and ACS groups.

Response�

Thank you for your suggestion. The author has modified the interpretation and discussion of the results by cautiously stating the limitations and clinical application prospects of the conclusions.

Minor Comment 3: The Data Availability Statement should be clarified. The manuscript indicates that data are available in a public repository, but no specific repository name, URL, or accession number is provided. Please provide explicit details to allow readers to access the underlying dataset in accordance with PLOS data-sharing requirements.

Response�

Thank you. This study used data from our site within the International Stroke Perfusion Imaging Registry (https://astn.net.au/inspire/). We had add the description in cover letter.

Dear reviewer 2:

Please find our revision for the above reference manuscript entitled: “Endovascular treatment based on computer tomography perfusion for basilar artery occlusion compared with anterior circulation stroke” for consideration for publishing in PLOS ONE as an original article.

We have revised our manuscript based upon the comments from the reviewer. We would like to take this opportunity to thank you and the reviewers for taking the time to review our submission, and for your thoughtful comments and suggestions to improve the quality of our manuscript.

We provide a point-by point response to the reviewers’ comments below.

This study, based on a case-control study, compared the outcomes of endovascular therapy (EVT) in patients with vertebrobasilar artery occlusion (BAO) versus those with anterior circulation stroke (ACS) using computed tomography perfusion (CTP). The results indicate that the efficacy of EVT in CTP-selected BAO patients is similar to that in ACS patients. BAO patients can also benefit from EVT initiated within 24 hours of symptom onset.

1.Did this study exclude patients with tandem lesions? If not, were subgroup analyses or sensitivity analyses performed for these patients to control for confounding effects?

Response:

Thank you for your question. There are only two tandem lesions patients in BAO group which combined with vertebral artery occlusion. All patients including BAO and ACS were reperfusion. So Our study did not exclude the patients with tandem lesions.

2.In the assessment based on computed tomography perfusion (CTP), were quantitative parameters such as cerebral blood flow (CBF) and cerebral blood volume (CBV) specifically included? Can these parameters be used for patient selection or as prognostic predictors?

Response:

Thank you for your question. These parameters were including to evaluate patients before EVT. The hypoperfusion volume was defined using a delay time (DT) threshold of >3 seconds. The ischemic core volume was calculated using a relative cerebral blood flow (rCBF) threshold of <30%.(Described in paragraph “Imaging protocol”). However we did not use these to patient selection or as prognostic predictors delectly. Maybe we could study the relationships between these quantitative parameters and clinical outcomes retrospectively.

3. Given that the time of admission or imaging detection for patients with posterior circulation infarction is often later than for those with anterior circulation occlusion, I would suggest that the authors perform a stratified analysis based on the time from symptom onset to randomization (or to EVT initiation) to better assess whether the treatment effect differs across different time windows.

Response:

Thank you for your suggestion. In our study, ‘two groups’ time of onset-door and onset-puncture for patients are similar (Table 2). So we did not perform a stratified analysis based on the time from symptom onset to randomization.

Attachment Submitted filename: Response to reviwer 2.doc

# Peer review: Decision Letter 1

**DOI:** 10.1371/journal.pone.0353204.r003

**Authors:** Aldobrando Broccolini, Aldobrando Broccolini

12 Jun 2026

PONE-D-26-14926R1

Endovascular treatment based on computer tomography perfusion for basilar artery occlusion compared with anterior circulation stroke – a case control study

PLOS One

Dear Dr. cai,

Thank you for submitting your manuscript to PLOS ONE. After careful consideration, we feel that it has merit but does not fully meet PLOS ONE’s publication criteria as it currently stands. Therefore, we invite you to submit a revised version of the manuscript that addresses the points raised during the review process.

Please note that one reviewer has raised additional issues that need to be properly addressed, along my personal editorial assessment that you can find below.

Please submit your revised manuscript by Jul 27 2026 11:59PM. If you will need more time than this to complete your revisions, please reply to this message or contact the journal office at plosone@plos.org. When you're ready to submit your revision, log on to https://www.editorialmanager.com/pone/ and select the 'Submissions Needing Revision' folder to locate your manuscript file.

Please include the following items when submitting your revised manuscript:

- A letter that responds to each point raised by the academic editor and reviewer(s). You should upload this letter as a separate file labeled 'Response to Reviewers'.
- A marked-up copy of your manuscript that highlights changes made to the original version. You should upload this as a separate file labeled 'Revised Manuscript with Track Changes'.
- An unmarked version of your revised paper without tracked changes. You should upload this as a separate file labeled 'Manuscript'.

If you would like to make changes to your financial disclosure, please include your updated statement in your cover letter. Guidelines for resubmitting your figure files are available below the reviewer comments at the end of this letter.

If applicable, we recommend that you deposit your laboratory protocols in protocols.io to enhance the reproducibility of your results. Protocols.io assigns your protocol its own identifier (DOI) so that it can be cited independently in the future. For instructions see: https://journals.plos.org/plosone/s/submission-guidelines#loc-laboratory-protocols. Additionally, PLOS ONE offers an option for publishing peer-reviewed Lab Protocol articles, which describe protocols hosted on protocols.io. Read more information on sharing protocols at https://plos.org/protocols?utm_medium=editorial-email&utm_source=authorletters&utm_campaign=protocols.

As the corresponding author, your ORCID iD is verified in the submission system and will appear in the published article. PLOS supports the use of ORCID, and we encourage all coauthors to register for an ORCID iD and use it as well. Please encourage your coauthors to verify their ORCID iD within the submission system before final acceptance, as unverified ORCID iDs will not appear in the published article. *Only*  the individual author can complete the verification step; PLOS staff *cannot*  verify ORCID iDs on behalf of authors.

We look forward to receiving your revised manuscript.

Kind regards,

Aldobrando Broccolini, MD, PhD

Academic Editor

PLOS One

Journal Requirements:

If the reviewer comments include a recommendation to cite specific previously published works, please review and evaluate these publications to determine whether they are relevant and should be cited. There is no requirement to cite these works unless the editor has indicated otherwise.

Additional Editor Comments:

The revised manuscript has been substantially improved and the authors have satisfactorily addressed the major concerns raised during the previous round of peer review. The study addresses a clinically relevant question and provides useful information regarding the imaging characteristics and outcomes of patients with basilar artery occlusion undergoing endovascular treatment.

However, one reviewer has raised additional comments during the current evaluation. These observations, together with my own editorial considerations, identify several remaining issues that should be addressed to further improve the scientific accuracy, methodological transparency, and interpretation of the findings.

Therefore, before the manuscript can be further considered, I would ask the authors to address the following points.

1. Study framing and title

The current title and several statements throughout the Abstract, Discussion, and Conclusions imply that the study evaluates the efficacy of CT perfusion-guided endovascular treatment. However, all included patients underwent CT perfusion imaging and no comparator group without perfusion imaging is available. Consequently, the study cannot determine whether CT perfusion guidance improves patient selection or clinical outcomes. The title and corresponding statements should therefore be revised to more accurately reflect the study design, namely a comparison of clinical characteristics, perfusion imaging findings, and outcomes between CTP-selected basilar artery occlusion and anterior circulation stroke patients undergoing EVT, rather than an evaluation of the effectiveness of a CTP-guided strategy.

2. Introduction and Discussion

The statement in the Introduction:

“Due to limited scientific evidence, the approaches to EVT for BAO remain heterogeneous due to the lack of data from randomized clinical trials.” should be removed or substantially revised, as it is no longer supported by the available evidence.

Similarly, the opening sentence of the Discussion: “Despite recent breakthroughs in the treatment of ischemic stroke caused by large artery occlusion (LAO), the optimal treatment for basilar artery occlusion (BAO) remains unclear, as BAO patients were excluded from recent randomized controlled trials.” should be removed, as it is factually incorrect. The ATTENTION and BAOCHE trials, together with BASICS and BEST, have established the benefit of EVT in appropriately selected BAO patients. The discussion should instead focus on the remaining uncertainties regarding imaging selection, perfusion assessment, and specific clinical subgroups.

3. Propensity score matching and statistical methods

The propensity score matching methodology should be incorporated into the Statistical Analysis section to improve the organization and transparency of the Methods.

Moreover, the manuscript describes the matched cohort as "well balanced," despite a substantial residual difference in baseline NIHSS score. Given that NIHSS is one of the strongest determinants of functional outcome and mortality, this imbalance deserves explicit discussion. Importantly, the known limitations of the NIHSS in posterior circulation stroke do not diminish the significance of this finding; rather, the persistence of a significantly higher NIHSS score in the BAO cohort despite the underrepresentation of posterior circulation symptoms suggests that these patients may have had an even greater neurological severity than reflected by the scale. The authors should clarify the rationale for excluding baseline NIHSS from the propensity score model, discuss the potential impact of this residual imbalance, and reconsider describing the matched cohort as "well balanced."

4. Outcome assessment

The Discussion should acknowledge that the modified Rankin Scale, although the standard functional endpoint in stroke trials, may incompletely capture clinically meaningful residual deficits frequently observed after posterior circulation stroke. Consequently, comparable mRS outcomes may underestimate differences in the actual burden of disability between posterior and anterior circulation stroke patients.

5. Applicability of CT perfusion parameters

Given the unique anatomical and hemodynamic characteristics of the posterior circulation, the Discussion would benefit from a brief consideration of the applicability and current evidence supporting conventional CT perfusion thresholds in basilar artery occlusion. The authors should clarify that the present study applies established perfusion parameters but does not aim to validate or optimize CTP thresholds for posterior circulation stroke.

6. Limitations

The current Limitations section is overly concise and should be substantially expanded. In addition to the retrospective design and the relatively small BAO cohort, the authors should explicitly discuss the absence of a non-CTP comparator group, the potential for residual confounding related to the persistence of baseline NIHSS differences after propensity score matching, and the implications of these limitations when interpreting the comparative effectiveness and safety of EVT between BAO and ACS.

7. Manuscript presentation

The overall presentation of the manuscript requires careful revision. The current version contains numerous formatting inconsistencies, including irregular punctuation and spacing, figure legends embedded within the main text, and other editorial artifacts that adversely affect readability. The manuscript should be carefully proofread to ensure that the text, figures, legends, and tables are presented in a clear and consistent manner.

To facilitate the editorial assessment of the revised version, please make sure that all modifications introduced in response to the present comments are clearly identified by highlighting throughout the manuscript.

[Note: HTML markup is below. Please do not edit.]

Reviewers' comments:

Reviewer's Responses to Questions

**Comments to the Author**

1. If the authors have adequately addressed your comments raised in a previous round of review and you feel that this manuscript is now acceptable for publication, you may indicate that here to bypass the “Comments to the Author” section, enter your conflict of interest statement in the “Confidential to Editor” section, and submit your "Accept" recommendation.

Reviewer #2: All comments have been addressed

**********

2. Is the manuscript technically sound, and do the data support the conclusions?

The manuscript must describe a technically sound piece of scientific research with data that supports the conclusions. Experiments must have been conducted rigorously, with appropriate controls, replication, and sample sizes. The conclusions must be drawn appropriately based on the data presented.

Reviewer #2: Partly

**********

3. Has the statistical analysis been performed appropriately and rigorously?

Reviewer #2: Yes

**********

4. Have the authors made all data underlying the findings in their manuscript fully available?

The PLOS Data policy requires authors to make all data underlying the findings described in their manuscript fully available without restriction, with rare exception (please refer to the Data Availability Statement in the manuscript PDF file). The data should be provided as part of the manuscript or its supporting information, or deposited to a public repository. For example, in addition to summary statistics, the data points behind means, medians and variance measures should be available. If there are restrictions on publicly sharing data—e.g. participant privacy or use of data from a third party—those must be specified.

Reviewer #2: Yes

**********

5. Is the manuscript presented in an intelligible fashion and written in standard English?

PLOS ONE does not copyedit accepted manuscripts, so the language in submitted articles must be clear, correct, and unambiguous. Any typographical or grammatical errors should be corrected at revision, so please note any specific errors here.

Reviewer #2: Yes

**********

6. Review Comments to the Author

Please use the space provided to explain your answers to the questions above. You may also include additional comments for the author, including concerns about dual publication, research ethics, or publication ethics. (Please upload your review as an attachment if it exceeds 20,000 characters)

Reviewer #2: This study compared the differences in perfusion imaging between basilar artery occlusion (BAO) and conventional anterior circulation stroke, highlighting the special application value and challenges of CTP in BAO. The following review comments are provided:

1.Due to the unique anatomical structure of the posterior circulation, are the widely used anterior circulation CTP parameters still applicable to patients with posterior circulation infarction?

2.The NIHSS itself has limitations in assessing common posterior circulation stroke symptoms (such as unsteady gait, dysphagia, and oculomotor disorders). In this study, although the BAO group had a higher baseline NIHSS score, the 90-day favorable outcome rate was not significantly worse than that of the anterior circulation group? Could the authors further explain or explore whether this phenomenon is attributable to the limitations of the NIHSS?

3.Patients with posterior circulation stroke often experience residual disabilities affecting quality of life, such as balance dysfunction, dysphagia, and dysarthria, which may not be adequately captured by the mRS. Therefore, using only the mRS to assess prognosis in posterior circulation stroke may underestimate the actual burden of disability.

**********

7. PLOS authors have the option to publish the peer review history of their article (what does this mean?). If published, this will include your full peer review and any attached files.

If you choose “no”, your identity will remain anonymous but your review may still be made public.

**Do you want your identity to be public for this peer review?**  For information about this choice, including consent withdrawal, please see our Privacy Policy.

Reviewer #2: No

**********

[NOTE: If reviewer comments were submitted as an attachment file, they will be attached to this email and accessible via the submission site. Please log into your account, locate the manuscript record, and check for the action link "View Attachments". If this link does not appear, there are no attachment files.]

To ensure your figures meet our technical requirements, please review our figure guidelines: https://journals.plos.org/plosone/s/figures

You may also use PLOS’s free figure tool, NAAS, to help you prepare publication quality figures: https://journals.plos.org/plosone/s/figures#loc-tools-for-figure-preparation.

NAAS will assess whether your figures meet our technical requirements by comparing each figure against our figure specifications.

# Author comment: Author response to Decision Letter 2

**DOI:** 10.1371/journal.pone.0353204.r004

17 Jun 2026

Dear reviewer 2:

Please find our revision for the above reference manuscript entitled: “Endovascular treatment based on computer tomography perfusion for basilar artery occlusion compared with anterior circulation stroke” for consideration for publishing in PLOS ONE as an original article.

We have revised our manuscript based upon the comments from the reviewer. We would like to take this opportunity to thank you and the reviewers for taking the time to review our submission, and for your thoughtful comments and suggestions to improve the quality of our manuscript.

We provide a point-by point response to the reviewers’ comments below.

1.Due to the unique anatomical structure of the posterior circulation, are the widely used anterior circulation CTP parameters still applicable to patients with posterior circulation infarction?

Thanks for your question. Given the unique anatomical and hemodynamic characteristics of the posterior circulation, the present study applies established perfusion parameters but does not aim to validate or optimize CTP thresholds for posterior circulation stroke.

2.The NIHSS itself has limitations in assessing common posterior circulation stroke symptoms (such as unsteady gait, dysphagia, and oculomotor disorders). In this study, although the BAO group had a higher baseline NIHSS score, the 90-day favorable outcome rate was not significantly worse than that of the anterior circulation group? Could the authors further explain or explore whether this phenomenon is attributable to the limitations of the NIHSS?

Thanks for your question. This phenomenon is closely related to the inherent defects of the NIHSS, which is a major contributing factor.

First, the NIHSS is primarily designed and validated for evaluating anterior circulation stroke. It assigns high scores to hemiplegia, aphasia and cortical dysfunction that are typical of anterior circulation lesions, while insufficiently weighting or even ignoring core manifestations of posterior circulation stroke including gait ataxia, dysphagia and oculomotor deficits. In patients with basilar artery occlusion (BAO), severe brainstem dysfunction can raise the baseline NIHSS score obviously, yet many of these high-scoring items reflect symptoms that have relatively good recovery potential after standard treatment.

Second, many disabling symptoms of posterior circulation stroke such as severe ataxia and persistent dysphagia are not fully reflected in the NIHSS. As a result, the baseline score overestimates the actual long-term functional impairment of BAO patients. Even with higher initial NIHSS scores, their actual neurological damage is not disproportionately severe, so the 90-day favorable outcome rate is comparable to that of the anterior circulation group.

Besides, other confounding factors cannot be ruled out: the diversity of collateral circulation in posterior circulation, differences in treatment timeliness and individual tolerance to therapy also affect clinical outcomes. In further studies, subgroup analysis focusing on NIHSS item composition and long-term functional scales (e.g. mRS) is recommended to quantitatively verify the impact of NIHSS limitations.

3.Patients with posterior circulation stroke often experience residual disabilities affecting quality of life, such as balance dysfunction, dysphagia, and dysarthria, which may not be adequately captured by the mRS. Therefore, using only the mRS to assess prognosis in posterior circulation stroke may underestimate the actual burden of disability.

Thanks for your question. You have raised a very valuable point. The modified Rankin Scale (mRS) indeed has obvious shortcomings when applied to posterior circulation stroke.

The mRS mainly focuses on overall daily living ability, mobility and self-care status. It cannot sensitively reflect subtle but persistent impairments including balance disturbance, mild dysphagia and dysarthria. These sequelae are highly prevalent in posterior circulation stroke survivors and severely impair their daily communication, eating and walking stability, yet they often correspond to relatively low mRS grades.

For this reason, relying solely on the mRS will underestimate the long-term disability and life impact of such patients. In further studies, combine specialized scales: for example, use ataxia scales, swallowing function scales and speech evaluation tools together with the mRS, so as to comprehensively evaluate the true functional status and disease burden of posterior circulation stroke patients.

We had added these sentence in discussions in revised manuscript.

Dear editor:

Please find our revision for the above reference manuscript entitled: “Endovascular treatment based on computer tomography perfusion for basilar artery occlusion compared with anterior circulation stroke” for consideration for publishing in PLOS ONE as an original article.

We have revised our manuscript based upon the comments from the editor. We would like to take this opportunity to thank you and the reviewers for taking the time to review our submission, and for your thoughtful comments and suggestions to improve the quality of our manuscript.

We provide a point-by point response to the reviewers’ comments below.

1. Study framing and title

The current title and several statements throughout the Abstract, Discussion, and Conclusions imply that the study evaluates the efficacy of CT perfusion-guided endovascular treatment. However, all included patients underwent CT perfusion imaging and no comparator group without perfusion imaging is available. Consequently, the study cannot determine whether CT perfusion guidance improves patient selection or clinical outcomes. The title and corresponding statements should therefore be revised to more accurately reflect the study design, namely a comparison of clinical characteristics, perfusion imaging findings, and outcomes between CTP-selected basilar artery occlusion and anterior circulation stroke patients undergoing EVT, rather than an evaluation of the effectiveness of a CTP-guided strategy.

Response:

Thanks for your suggestion. We have rewritten the title as “Perfusion imaging findings, and outcomes between CTP-selected basilar artery occlusion and anterior circulation stroke patients undergoing EVT”.

2. Introduction and Discussion

The statement in the Introduction:

“Due to limited scientific evidence, the approaches to EVT for BAO remain heterogeneous due to the lack of data from randomized clinical trials.” should be removed or substantially revised, as it is no longer supported by the available evidence.

Similarly, the opening sentence of the Discussion: “Despite recent breakthroughs in the treatment of ischemic stroke caused by large artery occlusion (LAO), the optimal treatment for basilar artery occlusion (BAO) remains unclear, as BAO patients were excluded from recent randomized controlled trials.” should be removed, as it is factually incorrect. The ATTENTION and BAOCHE trials, together with BASICS and BEST, have established the benefit of EVT in appropriately selected BAO patients. The discussion should instead focus on the remaining uncertainties regarding imaging selection, perfusion assessment, and specific clinical subgroups.

Thanks for your correction. We have deleted the sentence in revised manuscript.

3. Propensity score matching and statistical methods

The propensity score matching methodology should be incorporated into the Statistical Analysis section to improve the organization and transparency of the Methods.

Moreover, the manuscript describes the matched cohort as "well balanced," despite a substantial residual difference in baseline NIHSS score. Given that NIHSS is one of the strongest determinants of functional outcome and mortality, this imbalance deserves explicit discussion. Importantly, the known limitations of the NIHSS in posterior circulation stroke do not diminish the significance of this finding; rather, the persistence of a significantly higher NIHSS score in the BAO cohort despite the underrepresentation of posterior circulation symptoms suggests that these patients may have had an even greater neurological severity than reflected by the scale. The authors should clarify the rationale for excluding baseline NIHSS from the propensity score model, discuss the potential impact of this residual imbalance, and reconsider describing the matched cohort as "well balanced."

According to your request, the PSM in method section has been incorporated into the Statistical Analysis section.

The paragraph in the discussion “Consistent with previous studies comparing ACS and PCS [17-19], patients with BAO had higher baseline NIHSS scores and larger ischemic volume. Higher baseline NIHSS scores is partly due to the limitations of the NIHSS in assessing neurological deficits in patients with PCS, as it is highly weighted toward deficits occurring in ACS, such as aphasia and hemiparesis, while neglecting deficits such as unsteady gait, dysphagia, or oculomotor disorders[20]. Larger ischemic volume is partly due to limited collateral circulation in posterior circulation brain tissue[18].” explains why BAO has a high NIHSS score, which is a disease characteristic that cannot be well balanced, otherwise it would be difficult to include matched patients. The imbalance of this baseline feature and the relative balance of the results indicate that BAO may have gained greater benefits after this treatment.

4. Outcome assessment

The Discussion should acknowledge that the modified Rankin Scale, although the standard functional endpoint in stroke trials, may incompletely capture clinically meaningful residual deficits frequently observed after posterior circulation stroke. Consequently, comparable mRS outcomes may underestimate differences in the actual burden of disability between posterior and anterior circulation stroke patients.

Thanks for your correction. We have added the sentence “the mRS score, although the standard functional endpoint in stroke trials, may incompletely capture clinically meaningful residual deficits frequently observed after posterior circulation stroke. Consequently, comparable mRS outcomes may underestimate differences in the actual burden of disability between posterior and anterior circulation stroke patients.” in revised manuscript.

5. Applicability of CT perfusion parameters

Given the unique anatomical and hemodynamic characteristics of the posterior circulation, the Discussion would benefit from a brief consideration of the applicability and current evidence supporting conventional CT perfusion thresholds in basilar artery occlusion. The authors should clarify that the present study applies established perfusion parameters but does not aim to validate or optimize CTP thresholds for posterior circulation stroke.

Thanks for your correction. We have added the sentence “the present study applies established perfusion parameters but does not aim to validate or optimize CTP thresholds for posterior circulation stroke.” in revised manuscript.

“ 6. Limitations

The current Limitations section is overly concise and should be substantially expanded. In addition to the retrospective design and the relatively small BAO cohort, the authors should explicitly discuss the absence of a non-CTP comparator group, the potential for residual confounding related to the persistence of baseline NIHSS differences after propensity score matching, and the implications of these limitations when interpreting the comparative effectiveness and safety of EVT between BAO and ACS.

Thanks for your correction. We have rewritten the limitation as “This study has several important limitations. First, this was a single-center retrospective observational study, which may be subject to selection bias and unmeasured confounding. Second, the sample size of patients with BAO was relatively small compared with ACS group, which may limit the statistical power and generalizability of the results. Third, all patients included in this study underwent CTP imaging, and no control group treated without CTP guidance was available. Therefore, we cannot determine whether CTP-based selection improved patient eligibility, treatment decisions, or clinical outcomes compared with conventional non-perfusion imaging strategies. Fourth, despite the use of propensity score matching to balance baseline characteristics between groups, residual differences in baseline NIHSS scores persisted, which may introduce residual confounding that could not be fully adjusted for. This may affect the interpretation of the comparative effectiveness and safety of EVT between BAO and ACS patients. Finally, mRS as the primary functional outcome may incompletely capture posterior circulation–specific deficits such as dysphagia, ataxia, and dysarthria, which could lead to underestimation of the true disability burden in BAO patients.

These limitations should be considered when interpreting the comparative effectiveness and safety of EVT in patients with BAO versus ACS.”

7. Manuscript presentation

The overall presentation of the manuscript requires careful revision. The current version contains numerous formatting inconsistencies, including irregular punctuation and spacing, figure legends embedded within the main text, and other editorial artifacts that adversely affect readability. The manuscript should be carefully proofread to ensure that the text, figures, legends, and tables are presented in a clear and consistent manner.

Thanks for your correction. We have checked numerous formatting inconsistencies, including irregular punctuation and spacing, figure legends embedded in revised manuscript.

Attachment Submitted filename: revised to editor R2.docx

# Editor report: Decision Letter 2

**DOI:** 10.1371/journal.pone.0353204.r005

**Authors:** Aldobrando Broccolini, Aldobrando Broccolini, Aldobrando Broccolini

21 Jun 2026

Perfusion imaging findings, and outcomes between computed tomography perfusion selected basilar artery occlusion and anterior circulation stroke patients undergoing endovascular treatment

PONE-D-26-14926R2

Dear Dr. cai,

We’re pleased to inform you that your manuscript has been judged scientifically suitable for publication and will be formally accepted for publication once it meets all outstanding technical requirements.

Within one week, you’ll receive an e-mail detailing the required amendments. When these have been addressed, you’ll receive a formal acceptance letter and your manuscript will be scheduled for publication.

An invoice will be generated when your article is formally accepted. Please note, if your institution has a publishing partnership with PLOS and your article meets the relevant criteria, all or part of your publication costs will be covered. Please make sure your user information is up-to-date by logging into Editorial Manager at Editorial Manager® and clicking the ‘Update My Information' link at the top of the page. For questions related to billing, please contact billing support.

If your institution or institutions have a press office, please notify them about your upcoming paper to help maximize its impact. If they’ll be preparing press materials, please inform our press team as soon as possible -- no later than 48 hours after receiving the formal acceptance. Your manuscript will remain under strict press embargo until 2 pm Eastern Time on the date of publication. For more information, please contact onepress@plos.org.

Kind regards,

Aldobrando Broccolini, MD, PhD

Academic Editor

PLOS One

Additional Editor Comments (optional):

The Authors have addressed all the points raised by the Reviewer and the Academic Editor after the first round of review.

Reviewers' comments:

# Editor report: Acceptance letter

**DOI:** 10.1371/journal.pone.0353204.r006

**Authors:** Aldobrando Broccolini, Aldobrando Broccolini, Aldobrando Broccolini

PONE-D-26-14926R2

PLOS One

Dear Dr. Cai,

I'm pleased to inform you that your manuscript has been deemed suitable for publication in PLOS One. Congratulations! Your manuscript is now being handed over to our production team.

At this stage, our production department will prepare your paper for publication. This includes ensuring the following:

* All references, tables, and figures are properly cited

* All relevant supporting information is included in the manuscript submission,

* There are no issues that prevent the paper from being properly typeset

You will receive further instructions from the production team, including instructions on how to review your proof when it is ready. Please keep in mind that we are working through a large volume of accepted articles, so please give us a few days to review your paper and let you know the next and final steps.

Lastly, if your institution or institutions have a press office, please let them know about your upcoming paper now to help maximize its impact. If they'll be preparing press materials, please inform our press team within the next 48 hours. Your manuscript will remain under strict press embargo until 2 pm Eastern Time on the date of publication. For more information, please contact onepress@plos.org.

You will receive an invoice from PLOS for your publication fee after your manuscript has reached the completed accept phase. If you receive an email requesting payment before acceptance or for any other service, this may be a phishing scheme. Learn how to identify phishing emails and protect your accounts at https://explore.plos.org/phishing.

If we can help with anything else, please email us at customercare@plos.org.

Thank you for submitting your work to PLOS ONE and supporting open access.

Kind regards,

PLOS ONE Editorial Office Staff

on behalf of

Dr. Aldobrando Broccolini

Academic Editor

PLOS One
